# Detection of neuronal OFF periods as low amplitude neural activity segments

**DOI:** 10.1186/s12868-023-00780-w

**Published:** 2023-02-21

**Authors:** Christian D. Harding, Mathilde C. C. Guillaumin, Lukas B. Krone, Martin C. Kahn, Cristina Blanco-Duque, Christian Mikutta, Vladyslav V. Vyazovskiy

**Affiliations:** 1 Department of Physiology Anatomy and Genetics, Sir Jules Thorn Sleep and Circadian Neuroscience Institute, University of Oxford, Oxford, UK; 2 The Kavli Institute for Nanoscience Discovery, Oxford, UK; 3 Nuffield Department of Clinical Neurosciences, Sir Jules Thorn Sleep and Circadian Neuroscience Institute, University of Oxford, Oxford, UK; 4 Institute for Neuroscience, Department of Health Sciences and Technology, ETH Zürich, Schwerzenbach, Switzerland; 5 University Hospital of Psychiatry and Psychotherapy, University of Bern, Bern, Switzerland; 6 Centre for Experimental Neurology, University of Bern, Bern, Switzerland; 7 Picower Institute for Learning and Memory, Massachusetts Institute of Technology, Cambridge, USA; 8 Department of Brain and Cognitive Sciences, Massachusetts Institute of Technology, Cambridge, USA; 9 Translational Research Center, University Hospital of Psychiatry and Psychotherapy, University of Bern, Bern, Switzerland; 10 Private Clinic Meiringen, Meiringen, Switzerland

**Keywords:** Sleep, ON/OFF periods, Homeostasis

## Abstract

**Background:**

During non-rapid eye movement sleep (NREM), alternating periods of synchronised high (ON period) and low (OFF period) neuronal activity are associated with high amplitude delta band (0.5–4 Hz) oscillations in neocortical electrophysiological signals termed slow waves. As this oscillation is dependent crucially on hyperpolarisation of cortical cells, there is an interest in understanding how neuronal silencing during OFF periods leads to the generation of slow waves and whether this relationship changes between cortical layers. A formal, widely adopted definition of OFF periods is absent, complicating their detection. Here, we grouped segments of high frequency neural activity containing spikes, recorded as multiunit activity from the neocortex of freely behaving mice, on the basis of amplitude and asked whether the population of low amplitude (LA) segments displayed the expected characteristics of OFF periods.

**Results:**

Average LA segment length was comparable to previous reports for OFF periods but varied considerably, from as short as 8 ms to > 1 s. LA segments were longer and occurred more frequently in NREM but shorter LA segments also occurred in half of rapid eye movement sleep (REM) epochs and occasionally during wakefulness. LA segments in all states were associated with a local field potential (LFP) slow wave that increased in amplitude with LA segment duration. We found that LA segments > 50 ms displayed a homeostatic rebound in incidence following sleep deprivation whereas short LA segments (< 50 ms) did not. The temporal organisation of LA segments was more coherent between channels located at a similar cortical depth.

**Conclusion:**

We corroborate previous studies showing neural activity signals contain uniquely identifiable periods of low amplitude with distinct characteristics from the surrounding signal known as OFF periods and attribute the new characteristics of vigilance-state-dependent duration and duration-dependent homeostatic response to this phenomenon. This suggests that ON/OFF periods are currently underdefined and that their appearance is less binary than previously considered, instead representing a continuum.

**Supplementary Information:**

The online version contains supplementary material available at 10.1186/s12868-023-00780-w.

## Introduction

Slow waves, high amplitude oscillations in the delta frequency range (0.5–4 Hz) observed in electrophysiological signals are a characteristic of non-rapid eye movement sleep (NREM). A role of slow waves has been suggested in processes that are dependent on NREM sleep time, such as immune function and restoration of cognitive function [1]. Slow wave activity (SWA, spectral power in the delta frequency range) is a reliable index of sleep homeostasis [2] and local SWA homeostasis is correlated with improved motor learning task performance following sleep [3]. To help clarify the proposed involvement of slow waves in restorative processes and memory function, and to understand whether they are causally linked to these functions or rather simply a measurable output of an underlying process, it is vitally important that methods are developed which improve our understanding of the neurophysiology of slow waves.

Early studies, pioneered by Steriade et al. [4], showed that cortical slow waves during sleep depend on the synchronous hyperpolarization of cortical cells. The resulting periods of reduced neuronal activity, often referred to as OFF periods, are associated with depth positive/surface negative potentials in electrophysiological signals and alternate with periods of enhanced neuronal activity referred to as ON periods which are associated with depth negative/surface positive potentials [5]. Each slow wave cycle therefore corresponds to a single ON/OFF period alternation. It has been hypothesised that some of the functions ascribed to NREM sleep are in fact the result of neuronal inactivity during OFF periods [6]. For example, increases in neuronal activity resulting from sensory and optogenetic stimulation cause DNA double-strand breaks in neurons of mice [7]. These are repaired more rapidly during sleep than wakefulness, suggesting neuronal inactivity in NREM OFF periods may provide an opportunity for housekeeping functions such as repair of minor cellular damage [6, 9].

If it is indeed the dynamics of the synchronised neuronal activity that we are primarily interested in understanding, ON/OFF periods in local neuronal networks may provide a more direct measure of this behaviour than the slow waves it gives rise to. Despite this, far more attention has been given to detecting slow waves than ON/OFF periods. Slow wave detection methods have been developed based on amplitude and duration thresholds [10, 11], spectral frequency [12, 13] and using a neural network approach [14]. Whilst the number and quality of events detected as slow waves vary with method and implementation [15], these can at least be validated against a ‘gold standard’ manual scoring by experienced observers using tried and test criteria. Automated slow wave detection facilitates rapid processing of large data sets. Additionally, such methods have enabled the development of real time slow wave modulation, the therapeutic applications of which are currently being explored [16, 17].

Similarly, a range of methods have been devised to solve the problem of detecting ON/OFF period transitions. The simplest method is to apply amplitude and duration thresholds to spike trains: binary traces of high amplitude deflections (spikes) in single or multiunit activity signals [5, 18–21]. Though widely used both for offline and online detection of ON/OFF periods, this method is sensitive to the threshold used to define spikes which is often set using visual inspection and binarization results in the loss of a large amount of data. More sophisticated methods for detecting ON/OFF periods in continuous neuronal activity signals can broadly be classified as ‘threshold-crossing’ or ‘predictive’ algorithms. ‘Threshold-crossing’ algorithms work by processing the data until a bimodal distribution is obtained upon which a threshold is applied to separate data between ON and OFF periods [13, 22, 23]. ‘Predictive’ algorithms assume bimodality and assign data to one of either state based on the probability of a predictive model fitted to the data [24–26].

A common trend observed in the design of these detection algorithms is that they are built and tested upon a subset of data displaying clear slow waves [13] or ON/OFF oscillations [26] or acquired from anaesthetised animals where slow wave activity is generally more regular than during sleep [13, 22, 23, 25, 26]. Whilst it is likely that these methods will generalise to the case of sleep recordings, there may be advantages to designing a detection method on ‘noisy’ data characteristic of in vivo free-moving sleep recordings and that can be applied to the entire duration of chronic recordings used to study sleep behaviour. Another trend is to judge the performance of a detection method by comparing the output when applied to different signals recording the same neural activity (e.g. intra- vs extra- cellular, [13]) or with the output of other methods [25, 26]. As no method can yet be called the ‘gold standard’, this makes it challenging to judge the relative merit of methods and to assess the effect of optimisation steps within the pipeline (e.g. to remove short duration state transitions/interruptions).

An alternative design approach is to detect OFF periods that match the established characteristics of slow waves during spontaneous sleep and then infer ON periods retrospectively. OFF periods should occur predominantly, but not exclusively, in NREM sleep. SWA is highest and neuronal firing rate lowest in NREM sleep compared to wakefulness and rapid eye movement sleep (REM), which suggests that OFF periods are more likely to occur in this state [5]. However, slow waves are known to occur regularly during REM sleep [27] and occasionally during both inactive and active wakefulness [28–30]. By definition, OFF periods should be consistently associated with depth positive/surface negative deflections in the electroencephalogram (EEG) corresponding to the initiation of slow waves [5], and their duration should be positively correlated with slow wave amplitude [5]. Changes in the recruitment and decruitment of cortical neurons to ON/OFF periods respectively are associated with homeostatic dynamics and infraslow fluctuations of SWA, which may be a mechanism for synchronising neuronal activity across the cortex [5, 31]. Synchronization of OFF periods between brain regions should therefore be variable with widespread (global) OFF periods reflecting high decruitment of neurons and localised OFF periods reflecting low decruitment of neurons [32]. Finally, OFF periods should respond to changes in sleep pressure and circadian drive as a result of their homeostatic regulation [2].

The aim of this study was to determine if low activity is sufficient as a criterion to detect OFF periods in neuronal signals from freely behaving mice. To achieve this we designed a simple ‘threshold-crossing’ algorithm to identify a population of low amplitude (LA) segments in multiunit activity (MUA) recordings from freely behaving mice informed by the distribution of spiking amplitudes during NREM sleep and assessed whether these segments recapitulated the characteristics of OFF periods expected from the latter’s association with slow waves.

## Results

### Temporal features and state dependency

**Fig. 1:**
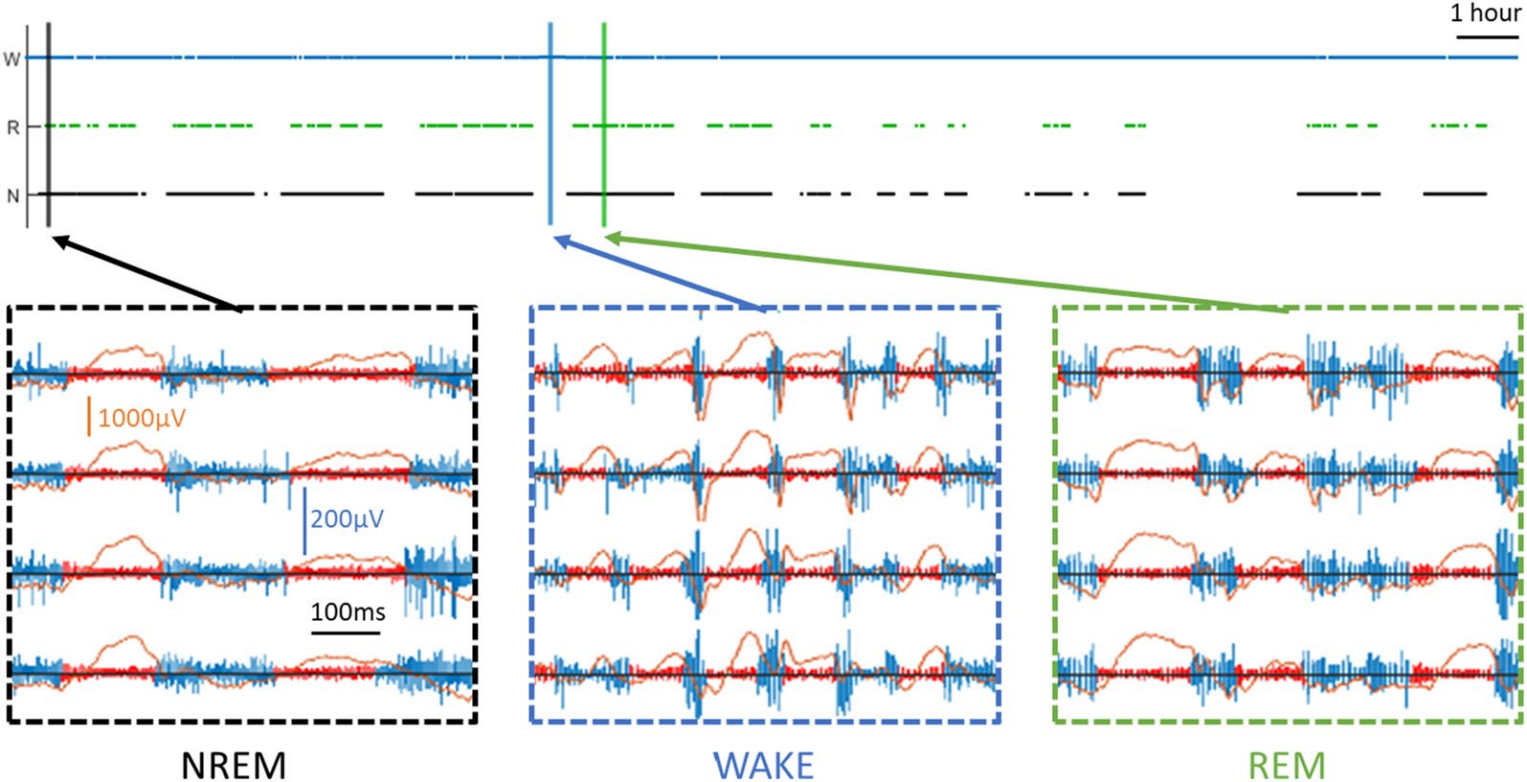
LA segments detected in all vigilance states. (top panel) 24-h hypnogram of vigilance states in a freely behaving mouse on a baseline recording day. Black = NREM sleep, green = REM sleep, blue = wakefulness. (bottom panels) Extracts of multiunit activity (MUA, vertical blue bars) and local field potential (LFP, horizontal orange line) from each vigilance state in four adjacent channels along a laminar probe implanted in motor cortex. Detected LA segments denoted in red. The top two channels are located in layer 3 whilst the bottom two channels are located in layer 5

**Fig. 2:**
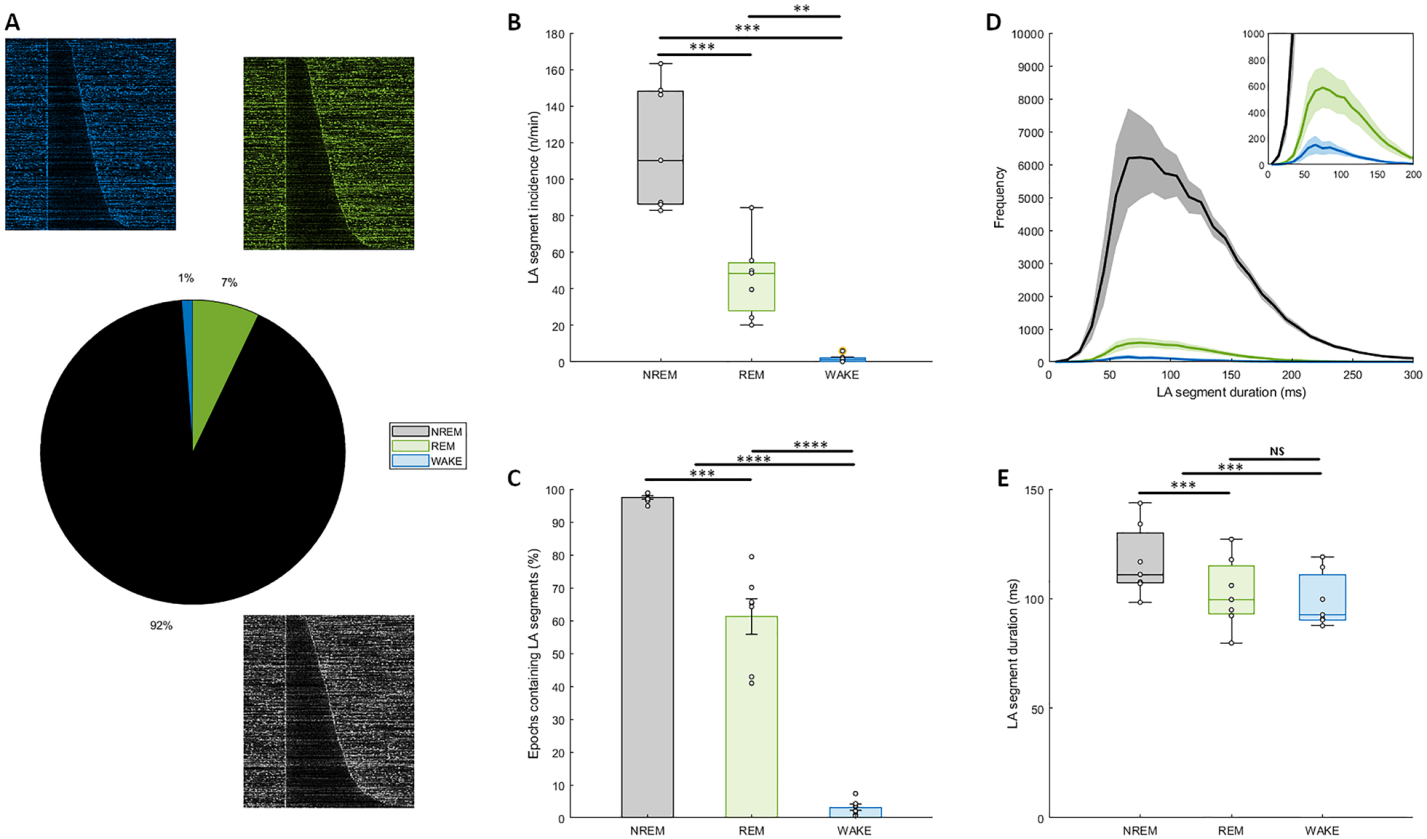
Temporal features of LA segments. **A** Global distribution of LA segments between vigilance states across study with examples of MUA amplitude during LA segments displayed for each state. Each figure shows 400 randomly selected LA segments sorted by duration. Segment time range = onset -100 ms to onset + 300 ms. Colour of each segment scaled to MUA amplitude (dark = low, colour = high). **B** The effect of vigilance state on the incidence of LA segments. Incidence values reported as number per minute of vigilance state. **C** The effect of vigilance state on the proportion of 4-s epochs containing an LA segment. **D** Frequency of LA segments as a function of duration for each vigilance state. Inset y-axis scaled to increase resolution of REM and WAKE states. **E** The effect of vigilance state on the duration of LA segments. Black/grey = NREM, green = REM, blue = WAKE. N = 7. Mean ± SEM. Significance of effects assessed using one-way repeated measures ANOVA followed by post-hoc pairwise t-tests with Bonferroni correction (*P < 0.05; **P < 0.01; ***P < 0.001; ****P < 0.0001)

LA segments were detected in all vigilance states in different cortical layers (Fig. 1). To characterise the temporal features of LA segments and compare their properties across vigilance states, we detected LA segments in MUA recordings from a representative layer 5 channel of motor cortex on the baseline (BL) day. We first looked at whether LA segments are more common in NREM sleep than other vigilance states. The majority of LA segments were detected in NREM sleep (72547.00 ± 9164.22, 91.70 ± 1.44%) and REM sleep (5675.43 ± 1405.64, 7.11 ± 1.46%) with a small proportion detected in WAKE (1130.29 ± 437.50, 1.20 ± 0.34%) (Fig. 2A). LA segment onset in all states is associated with a characteristic sharp drop in MUA amplitude (Fig. 2A). There was a significant effect of state on LA segment incidence (Fig. 2B, one-way repeated measures ANOVA, F(2,12) = 68.92, p < 0.05) with LA segments occurring most frequently in NREM sleep and least frequently in WAKE. There was also a significant effect of state on the proportion of 4-s epochs containing at least one LA segment (Fig. 2C, one-way repeated measures ANOVA, F(1.02,6.11) = 286.79, p < 0.05) with the vast majority of NREM epochs (97.37 ± 0.56%), the occasional WAKE epoch (3.20 ± 0.87%) and over half of REM epochs (61.19 ± 5.35%) containing an LA segment. These findings show that LA segments are preferentially, but not exclusively, associated with NREM sleep as has previously been reported for OFF periods [5, 27–30]. We then looked at the duration of LA segments to see how this compared with previously reported OFF period descriptions. LA segment durations ranged from 8 ms to greater than 1 s but on average lasted 116.26 ± 2.16 ms. This is slightly shorter than a previous description of OFF periods in mice of a comparable age (134 ms, [18]), however this was not unexpected considering the comparison is with a detection method targeting the long duration OFF periods. Thus, this finding does not preclude the possibility that LA segments are OFF periods. The distribution of LA segments was strongly positively skewed during all states (1.54 ± 0.18, Fig. 2D). The distributions were leptokurtic for WAKE (kurtosis = 5.16) and REM (3.68) LA segments, such that long LA segments occurred more frequently than in a standard Gaussian distribution, but there was no excess kurtosis in the NREM sleep distribution (3.00). LA segments were longer in NREM sleep (116.89 ± 6.11 ms) than either REM sleep (102.46 ± 6.06 ms) or WAKE (99.18 ± 4.77 ms) states in which LA segment durations were similar (Fig. 2E, one-way repeated measures ANOVA, F(2,12) = 34.48, p < 0.05) though the absolute difference was small (ca. 15 ms). Whilst the relative duration of OFF periods in different states has not been reported, this does mirror the finding that REM sleep slow waves are described as smaller than those occurring in NREM sleep in reference to their lower amplitude, which is correlated with OFF period duration [5, 27].

### Association with LFP

**Fig. 3:**
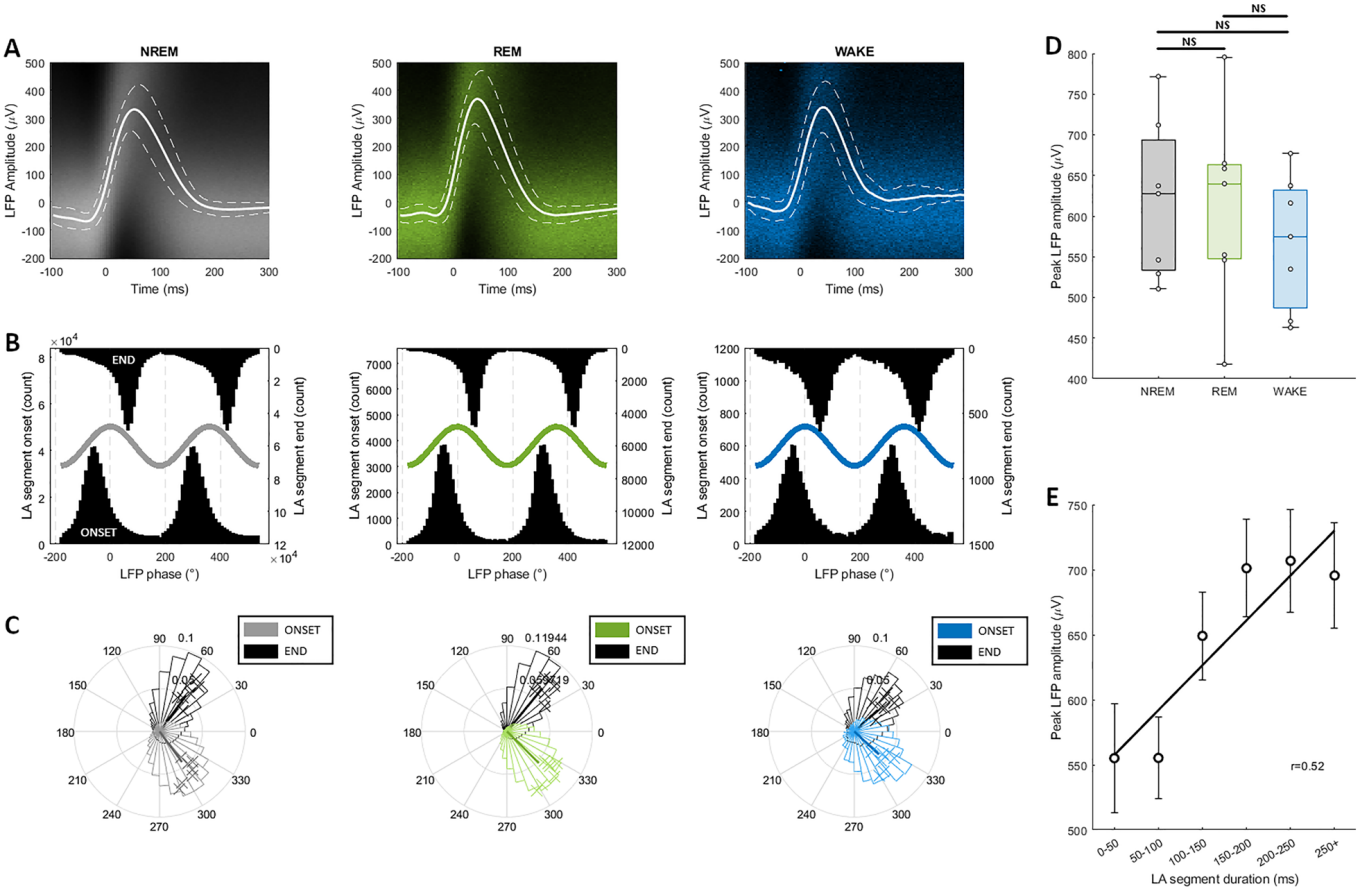
MUA LA segments associated with slow-wave activity in LFP. **A** Layer 5 LFP corresponding with LA segments in each vigilance state. Composite image of LFP traces converted to heatmap (higher colour saturation = higher density) overlaid with mean ± SEM. Segment time range = onset -100 ms to onset + 300 ms. **B** Global distributions of the LFP (2–6 Hz) phase corresponding to LA segment ONSET (bottom) and END (top) in each vigilance state. Example of a sinusoid wave overlaid for visualization purposes only. **C** Distribution of preferred LFP (2–6 Hz) phase corresponding to LA segment ONSET and END in each vigilance state (proportion) with mean resultant vector. **D** Effect of state on peak amplitude in LFP corresponding to LA segments. **E** Relationship between LA segment duration and peak amplitude of corresponding LFP. Least-squares regression line and significant Pearson correlation coefficient shown. Black/grey = NREM, green = REM, blue = WAKE. N = 7. Mean ± SEM. Significance of effects assessed using one-way repeated measures ANOVA followed by post-hoc pairwise t-tests with Bonferroni correction (*P < 0.05; **P < 0.01; ***P < 0.001; ****P < 0.0001)

Evidence suggests that OFF periods coincide with local field potential (LFP) slow waves [4, 5]. If LA segments represent OFF periods, we would expect a similar association. We first extracted a 400 ms window of LFP for each LA segment in a representative layer 5 channel during baseline sleep from onset -100 ms to onset + 300 ms and calculated the average LFP signal during LA segments during each vigilance state (Fig. 3A). In each state, LA segments were associated with a positive deflection of the LFP which coincides with segment onset. This deflection lasts around 150 ms, which if considered a half wave suggests a frequency of ~ 3 Hz, within the delta frequency range. This deflection was bounded by slight negative deflections. These features are consistent with LA segments being time locked to LFP slow waves. To confirm this, we performed a phase analysis to determine the preferred phase of LA segment onset and end in the delta frequency range of the LFP (Fig. 3B, C). LA segment onset preferentially occurred at ~ 310° (NREM: 306.26 ± 1.49°, REM: 315.31 ± 1.60°, WAKE: 316.84 ± 1.52°), coinciding with the rising limb of delta oscillations. LA segment end preferentially occurred at ~ 50° (NREM: 53.50 ± 0.97°, REM: 50.60 ± 1.24°, WAKE: 42.17 ± 1.43°), coinciding with the falling limb of delta oscillations. In all states, onset and end phase distributions were significantly different (NREM: Watson-Williams, F(1,12) = 487.39, p < 0.05, REM: Watson-Williams, F(1,12) = 286.79, p < 0.05, WAKE: Watson-Williams, F(1,12) = 238.83, p < 0.05).

Finally, we looked at how time locked peak LFP amplitude changed with vigilance state or LA segment duration. We found a significant effect of vigilance state on peak LFP amplitude (F(2,12) = 3.97, p = 0.047) however post-hoc pairwise t-tests did not reveal a significant difference in mean LFP amplitude between any states (Fig. 3D). This suggests that LA segments in all states were associated with similar amplitude slow waves. It has been reported that slow wave amplitude increases as a function of OFF period duration [32]. In agreement with this, we found that LA segment duration was positively correlated with peak LFP amplitude (Fig. 3E, r = 0.52, p < 0.05).

### Homeostatic regulation

**Fig. 4:**
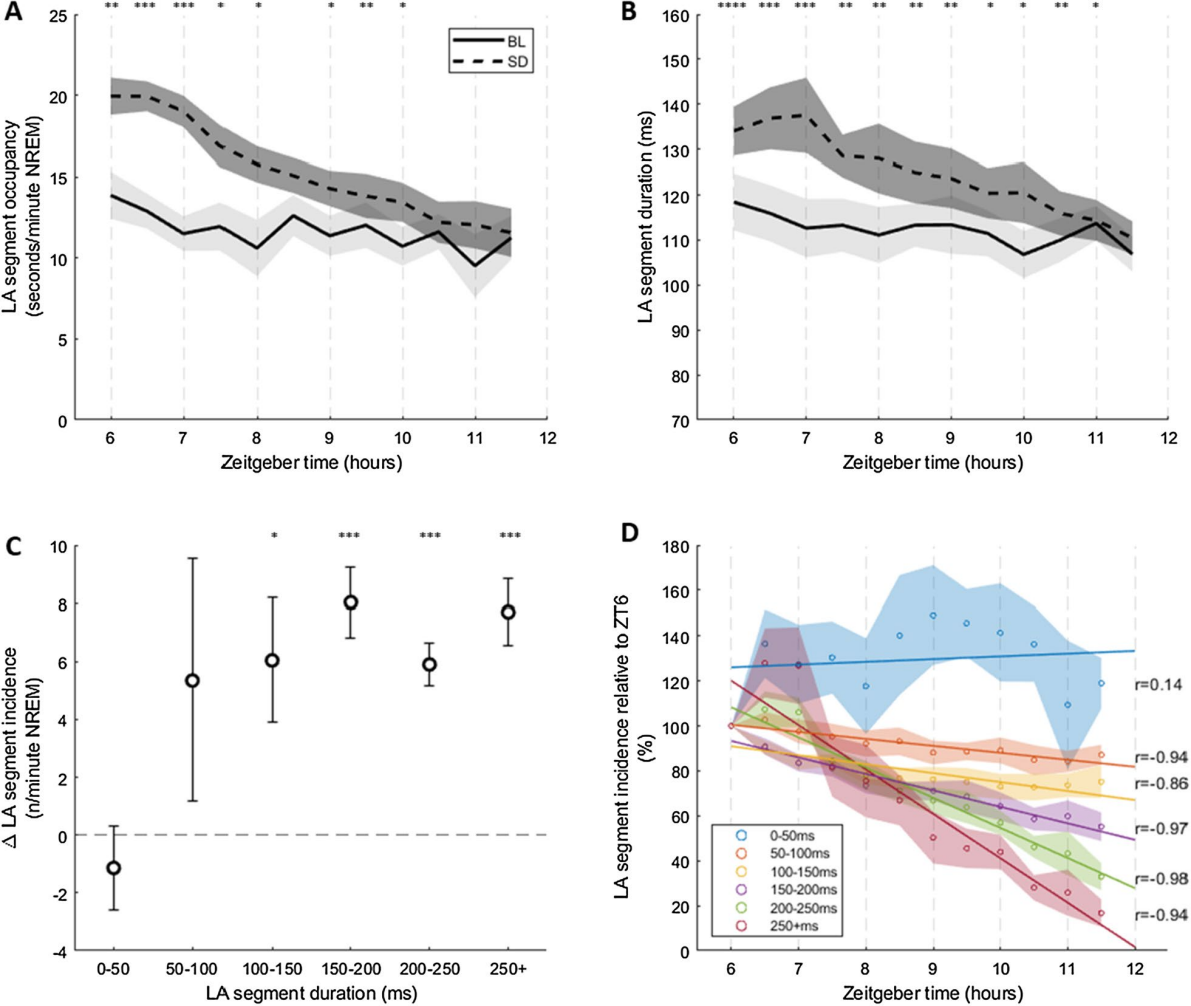
LA segments are homeostatically regulated. **A** Relationship between LA segment occupancy and time during NREM sleep between ZT6 and ZT12 on baseline (BL) and sleep deprivation (SD) days (30 min bins). Occupancy values reported as seconds of LA segments per minute of NREM. **B** Change in LA segment duration during NREM sleep between ZT6 and ZT12 on BL and SD days (30 min bins). **C** Relationship between LA segment duration and the change in LA segment incidence between BL and SD days during the 1st hour after sleep deprivation. A change > 0 describes an increase in incidence with the sleep deprivation treatment. **D** Relationship between LA segment incidence and time during NREM sleep between ZT6 and ZT12 on the sleep deprivation day as a function of LA segment duration. Circles denote mean incidence and lines show least-square regression for each duration category. N = 7^i^. Mean ± SEM. Significance of effects assessed using two-way repeated measures ANOVA followed by post-hoc pairwise t-tests with Bonferroni correction (*P < 0.05; **P < 0.01; ***P < 0.001; ****P < 0.0001). Only significant Pearson correlation coefficients shown. ^i^ One animal lacked LA segments < 50 ms for at least one time bin so was not included in the analysis for this group of segments in D

The build up and subsequent release of sleep pressure resulting from homeostatic regulation has two expected outcomes on slow waves and presumably OFF periods: slow wave activity should decrease over the course of the inactive period and slow wave activity should be higher during sleep after sleep deprivation [2]. To assess whether these features apply to LA segments, we analysed 6 h of spontaneous activity during the second half of the light phase (ZT6–ZT12) prior to which animals were undisturbed (baseline spontaneous activity, BL) or were kept awake by providing novel objects to explore (sleep deprivation, SD) for 6 h between ZT0–ZT6 on consecutive days. As in the previous sections, a single layer 5 channel was used to represent each animal. There was a significant effect of prior sleep–wake history on occupancy time (Fig. 4A, two-way repeated measures ANOVA, F(1,5) = 27.60, p < 0.05) and duration (Fig. 4B, two-way repeated measures ANOVA, F(1,4) = 48.14, p < 0.05) of LA segments with post-hoc pairwise t-tests confirming a significant increase in the both metrics between ZT6-ZT8.5 after SD. There was a significant effect of zeitgeber time on occupancy time (Fig. 4A, two-way repeated measures ANOVA, F(11,55) = 11.80, p < 0.05) and duration (Fig. 4B, two-way repeated measures ANOVA, F(11,44) = 7.04, p < 0.05) of LA segments with both metrics decreasing as a function of time. Finally, there was a significant interaction between prior sleep–wake history and zeitgeber time on occupancy time (Fig. 4A, two-way repeated measures ANOVA, F(11,55) = 3.73, p < 0.05) and duration (Fig. 4B, two-way repeated measures ANOVA, F(1,44) = 6.85, p < 0.05). To understand this further we performed post-hoc linear regressions for each condition separately. The regression of zeitgeber time against LA segment occupancy time was significant for the SD condition (linear regression model, F(1,82) = 71.756, p < 0.05) but not for the BL condition (linear regression model, F(1,81) = 3.74, p > 0.05). Similarly the regression of zeitgeber time against LA segment duration was significant for the SD condition (regression model, F(1,82) = 23.91, p < 0.05) but not for the BL condition (linear regression model, F(1,80) = 2.54, p > 0.05). To establish whether the absence of a homeostatic decrease in LA segment metrics for the BL condition was simply due to the animals having already paid the majority of their sleep debt by ZT6, we repeated regression analysis on data from the entire light period (ZT0-12) and indeed found significant regressions of zeitgeber time against LA segment occupancy (Additional file 1: Figure S1A, linear regression model, F(1165) = 14.28, p < 0.05) and duration (Additional file 1: Figure S1B, linear regression model, F(1164) = 10.97, p < 0.05).

OFF periods are usually portrayed as an all or none phenomenon. Thus OFF period duration would not be expected to have a significant effect on a feature such as homeostatic regulation. To assess whether this is true for LA segments, we binned segments in 5 groups based on duration and looked at incidence as a marker of homeostatic regulation. We found that there was a significant increase in LA segment incidence during the first hour after sleep deprivation as compared to spontaneous activity for segments of 100-150 ms (Fig. 4C, Bonferroni adjusted paired T-test, t(6) = − 2.81, p < 0.05), 150-200 ms (Fig. 4C, Bonferroni adjusted paired T-test, t(6) = − 6.67, p < 0.05), 200-250 ms (Fig. 4C, Bonferroni adjusted paired T-test, t(6) = − 8.06, p < 0.05) and 250 + ms (Fig. 4C, Bonferroni adjusted paired T-test, t(6) = − 6.69, p < 0.05) in duration. There was no significant change in incidence for LA segments < 50 ms or 50-100 ms in duration, though incidence of 50–100 ms LA segments varied considerably between subjects with 5/7 animals showing an increase of > 5 segments per minute following sleep deprivation. Furthermore, whilst the incidence of LA segments > 50 ms in duration was negatively correlated with time (Fig. 4D) in the 6 h following sleep deprivation, there was no correlation for segments < 50 ms. We noted that the magnitude of relative decrease in incidence over time appeared to increase with LA segment duration (Fig. 4D). To confirm this, we fit a linear regression model to the distribution of relative LA segment incidence to zeitgeber time for each LA segment duration category. Except for segments < 50 ms in duration, the regression of zeitgeber time against LA segment incidence was significant across all time bins (see Additional file 1: Figure S2). Furthermore, the estimate for the predictor variable (i.e. zeitgeber time) became increasingly negative with increasing LA segment duration (β = − 3.11 (50–100 ms) < − 3.99 (100–150 ms) < − 7.33 (150–200 ms), − 13.41 (200–250 ms) < − 19.75 (250 + ms)). Overall, the homeostatic response of LA segments does appear to depend on their duration, contrary to the expectation of OFF periods being an all or none phenomenon.

### Interchannel coherence

**Fig. 5:**
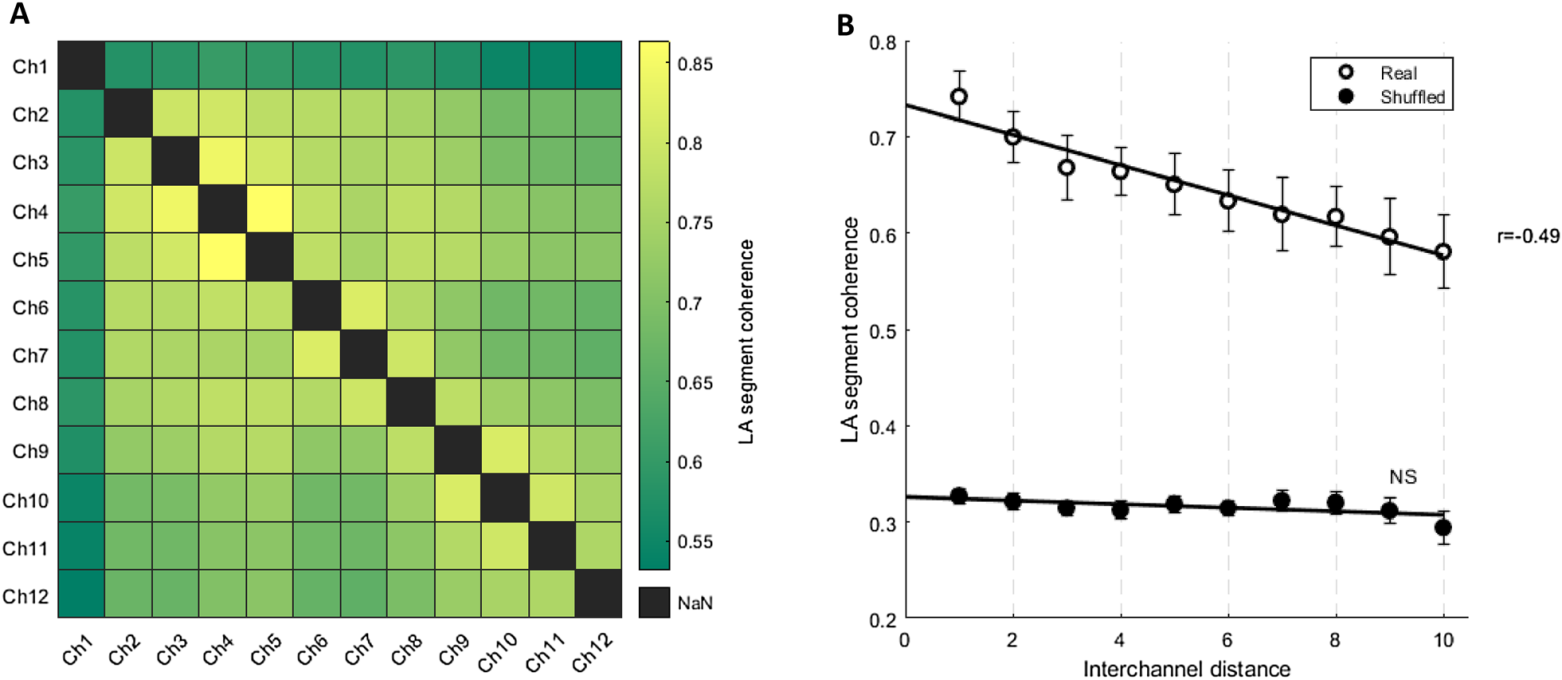
LA segments have both local and global dynamics. **A** Example temporal coherence matrix between 12 channels arranged by depth for one animal (green = low coherence, yellow = high coherence). Temporal coherence reported as the average proportion of time spent in the low amplitude state during which both channels are synchronously in a low amplitude state (see Methods). **B** Relationship between the temporal coherence of LA segments in pairs of channels and the distance between those channels along the laminar probe for both original and surrogate channels. N = 7. Mean ± SEM. Least-squares regression lines and significant Pearson correlation coefficients shown. There were insufficient data points to include the maximum interchannel distance (11)

OFF periods are both a global and a local phenomenon. To investigate whether this was also true of our LA segments, we determined the temporal coherence between channels separated by different distances along the laminar probe (i.e. different depths within motor cortex) and compared coherence at different interchannel distances (Fig. 5A, Additional file 1: Figures S3, S4). Coherence was calculated between all LA segments detected on the baseline day. Coherence was generally highest along the diagonal, that is, between adjacent channels. Although only channels with neural activity (i.e. spikes) as determined by visual inspection were retained, OFF periods in superficial channels were not always coherent with other channels (Fig. 5A, Ch1). Thus whilst our method still detects OFF periods, reliability may be lower for superficial channels. To determine whether any coherence found was greater than chance, we generated surrogate channels by randomly shuffling LA and non-LA segments from the original channels (see Methods). To avoid vigilance state-dependent effects we only used data from NREM sleep for coherence analysis and shuffling. There was a significant effect of channel type (Fig. 5B, two-way repeated measures ANOVA, F(1,6) = 109.51, p < 0.05), which suggests the LA segments in original channels, which have a higher mean coherence across all interchannel distances, are more synchronous than LA segments in surrogate channels. This finding highlights the generalised synchronicity of OFF periods across laminar layers. Furthermore, there were significant effects of interchannel distance (Fig. 5B, two-way repeated measures ANOVA, F(9,54) = 26.75, p < 0.05) and interchannel distance * type interaction (Fig. 5B, two-way repeated measures ANOVA, F(9,54) = 31.01, p < 0.05). We used Pearson correlation to interrogate the interaction term further and found that coherence was negatively correlated with original interchannel distance (Fig. 5B, r = − 0.49, p < 0.05) but was not correlated with surrogate interchannel distance. This suggests that in terms of LA segment incidence, neighbouring channels are more synchronous than distant channels for original channels but not for surrogate channels where channel pairs are equally synchronous across the range of interchannel distances. This is consistent with global neocortical OFF periods displaying subtle layer dependent effects (i.e. localised dynamics).

## Discussion

Previous attempts to identify OFF periods in high frequency neural activity have used methods of varying complexity and levels of processing to fit their results to co-occur with slow waves. Here we show that a population of low amplitude segments can be extracted from high frequency neural activity without prior knowledge of concomitant LFP activity which fit the expected characteristics of OFF periods.

LA segments present as brief reductions in MUA amplitude, primarily during NREM sleep but also appear in REM sleep and WAKE. LA segments usually last approximately 100 ms but duration is variable with many segments upwards of 1 s occurring. LA segments are associated with a positive deflection in deep neocortical LFP (layer 5) characteristic of slow wave initiation. Furthermore, LA segment onset and end times are phase locked to the LFP in the delta range where slow waves occur. The duration and total occupancy time per minute of NREM sleep of LA segments decreases throughout the inactive period of mice during spontaneous activity (Additional file 1: Figure S1) and after sleep deprivation (Fig. 4) whilst the absolute magnitude of both metrics is increased by sleep deprivation, consistent with expectations of a phenomenon regulated by sleep homeostasis [33]. Finally, LA segments are temporally synchronised across neocortical laminar layers at both a global and local scale. Together, these findings strongly suggest that LA segments represent OFF periods.

This finding is important for two key reasons. First, we extend the work of previous studies showing that OFF periods can be detected based on high frequency neural activity amplitude alone without reference to slow waves (e.g. [23]) by applying this methodology to recordings from freely behaving mice and by showing that it can be leveraged to detect OFF periods in all vigilance states. This permits detection of OFF periods in cases where the LFP is uninformative and opens up the possibility of studying LFP slow waves and MUA OFF periods as separate measures of the phenomenon of synchronised neuronal silencing events, each providing unique information at different scales of integration across brain regions. The MUA-based local assessment of OFF periods will be of particular importance for advancing the understanding of layer-specific dynamics in neocortex because LFP signals are influenced by volume conductance from adjacent layers and neighbouring brain regions. Second, we provide a simple method for detecting OFF periods that can be implemented as part of an easily accessible toolbox. This will allow for greater accessibility of OFF period analyses in electrophysiological sleep research and will help foster the growing interest in ‘local sleep’ effects in behavioural neuroscience.

### Homeostatic regulation of LA segments depends on segment duration

Unexpectedly, we found that the duration of LA segments had an effect on their homeostatic response. LA segments < 100 ms in duration showed a different homeostatic response to segments > 100 ms in duration. LA segments of 50–100 ms occurred more frequently immediately following sleep deprivation than after a few hours of recovery sleep, consistent with homeostatic regulation. However, they showed no increase in incidence after sleep deprivation compared with the same subjective hour on a day without prior sleep deprivation. LA segments < 50 ms showed neither a homeostatic decrease in incidence after sleep nor an increase after sleep deprivation. Most importantly, longer segments showed a steeper decrease in incidence following sleep deprivation than shorter segments, evidence of a greater response to a build-up of sleep pressure.

One explanation of these results is that short LA segments do not represent OFF periods as they are classically described. This may best explain the absence of a clear homeostatic response in the shortest LA segments (< 50 ms). Therefore, we suggest that these short LA segments are not consistent with current descriptions of OFF periods and that, as others have done previously [24], a minimum duration of 50 ms should be introduced to remove brief decreases in MUA amplitude that do not behave in a similar way to longer decreases commonly identified as OFF periods. However, this explanation does not account for the mixed results for 50–100 ms LA segments and the graded intra-day homeostatic response of LA segments by duration. Two hypotheses would explain this result. First, the proportion of LA segments that represent functionally relevant OFF periods as opposed to transient decreases in MUA amplitude may increase with OFF period duration. As such, the longest LA segments may show the strongest homeostatic response as the effect is less diluted by non-homeostatically regulated noise. Second, our findings are evidence that OFF periods and their associated dynamics lie on a continuum depending on the strength of neuronal recruitment and therefore duration. Greater recruitment leads to longer OFF periods which display a stronger homeostatic response to sleep pressure, either spontaneously generated or via sleep deprivation. We recognise that both hypotheses may fit the data, however we suggest that the latter may be more likely considering the abundant evidence of other OFF period-like behaviour for LA segments.

### LA segment characteristics show state dependency

As part of our validation of LA segments as OFF periods, we chose to differentiate between LA segments occurring in different vigilance states. As expected, LA segments occurred most frequently in NREM sleep but also occurred in the majority of REM sleep epochs and occasionally during wakefulness. Furthermore, LA segments had a similar LFP profile independent of vigilance states suggesting all were associated with slow waves. Less expected was the finding that LA segments during NREM sleep were longer than LA segments in REM sleep and wakefulness. This could suggest that OFF periods during wakefulness and REM sleep do not achieve the same recruitment of neurons to the OFF state than in NREM sleep. Wakefulness and REM OFF periods may be more localised as wakefulness and REM sleep are more ‘active’ states and as such the neuromodulatory milieu disrupts the formation of synchronous activity.

## Conclusion

We provide strong evidence that OFF periods can be detected by clustering together cortical multiunit activity segments of similarly low amplitude of extracellularly recorded neuronal spiking. These low amplitude segments show many characteristics expected of OFF periods, including NREM predominance, a strong association with LFP slow waves, sleep homeostasis and temporal coherence across cortical layers. Furthermore, we find that the incidence of longer LA segments respond more strongly to sleep pressure than short LA segments and that LA segments are longer in NREM sleep than REM sleep or wakefulness. These vigilance-state- and duration-dependent effects were not previously described for OFF periods but these findings may represent additional OFF period features that have either been overlooked or are only revealed with multiunit activity amplitude-only detection methods.

## Methods

All experiments were carried out in accordance with the UK Animals (Scientific Procedures) Act of 1986 and in compliance with the Animal Research: Reporting In Vivo Experiments (ARRIVE) guidelines.

### Surgery and electrode implantation

Adult male wild type C57BL/6 mice (n = 7, internally sourced from Biomedical Services at the University of Oxford, 125 ± 8 d old at baseline recording) underwent cranial surgery to record electroencephalography (EEG), electromyography (EMG), local field potential (LFP) and multiunit activity (MUA) as previously described [18]. Briefly, under ~ 2–3% isoflurane anaesthesia and aseptic conditions, stainless steel screws were implanted epidurally over frontal and occipital cortical areas and referenced to a third screw implanted over the cerebellum. Stainless steel wires were implanted into the nuchal muscle to record EMG. A 16-channel laminar probe (NeuroNexus Technologies Inc., Ann Arbor, MI, USA; model: A1 × 16–3 mm-100-703-Z16) was implanted in left primary motor cortex (AP + 1.1 mm; ML − 1.75 mm; rotated 15° in the AP axis towards the side of the implant) to perform intracortical recordings (LFP and MUA as described in [19]). The entire 3 mm length was inserted gradually into the tissue under both stereotactic and microscopic control until the most superficial electrode was approximately 50 µm under the cortical surface. The implantation site was then sealed with the silicone elastomer Kwik-Sil® (World Precision Instruments Inc., Sarasota, FL, USA) and the probe was referenced to the cerebellar skull screw. Depth and cortical layer of channels were subsequently determined by histology assessment (for histological methodology see [19]). Briefly, the position of the laminar implant was determined using a DiL (Thermo Fisher Scientific) fluorescence membrane stain and the depth of the laminar implant was assessed by measuring the distance between the cortical surface and tissue microlesions generated by applying 10 mA of direct current for 25 s to each respective channel using a NanoZ device (White Matter LLC). Each animal was also implanted with a bipolar concentric electrode (PlasticsOne Inc., Roanoke, VA, USA) in the right primary motor cortex, anterior to the frontal EEG screw in relation to a separate study (as described in [20]). Screw electrodes were attached to an 8-pin surface mount connector (8415-SM, Pinnacle Technology Inc, KS) whilst the laminar probe was attached to a ZIF-Clip® 16 channel headstage (Tucker-Davis Technologies Inc., Alachua, FL, USA) and both affixed to the skull with dental cement (Associated Dental Products Ltd, Swindon, UK).

### Electrophysiological signal acquisition

All signals were first passed to a PZ-5 pre-amplifier (Tucker-Davis Technologies Inc., Alachua, FL, USA). A 128-channel RZ-2 Neurophysiological Recording System (Tucker-Davis Technologies Inc., Alachua, FL, USA) was then used to acquire tethered electrophysiological recordings. EEG and EMG signals were continuously sampled at 305 Hz and bandpass filtered between 0.1–100 Hz. Signals were then downsampled offline to 256 Hz via spline interpolation. Laminar probe channel signals were sampled at 25 kHz. Two signals were extracted from the laminar probe channels: decimated multiunit activity (MUA) and local field potential (LFP). Multiunit activity is the high frequency component of neural activity that contains the spiking of multiple neurons within the vicinity of an electrode. Decimation is a process for downsampling the MUA whilst retaining spiking activity by storing only the highest amplitude value, either negative or positive, recorded during a set time period. This means that if multiple neurons spike during that period, only the largest is stored, thus the majority of spikes in the decimated signal will originate from nearby neurons. The resulting signal will therefore have a high amplitude when nearby neurons are spiking and a low amplitude during periods of quiescence or when distant neurons are spiking. MUA was generated by bandpass filtering the laminar signals between 300 Hz and 5 kHz then decimating to 498 Hz by splitting the signal into segments of ~ 50 samples and storing the maximum/minimum amplitude of alternating segments as integers. LFP was generated by zero-phase distortion bandpass filtering the laminar signal between 0.1 and 100 Hz and downsampling to 256 Hz via spline interpolation. All offline manipulations and analyses were performed using MATLAB (version R2020a; The MathWorks Inc, Natick, MA, USA). Prior to vigilance state scoring, signals were transformed into European Data Format as previously reported (see [30]).

### Experimental design and recording procedure

For sleep recordings, animals were individually housed in sound-attenuated and light-controlled Faraday chamber cages (Campden Instruments, Loughborough, UK) with ad libitum food and water. A 12:12 h light/dark cycle (lights on at 9 am = ZT0, light levels 120–180 lx) was implemented, temperature maintained at around 22 ± 2 °C, and humidity kept around 50 ± 20%. Animal were given at least three days post-surgery to acclimatize before two recording days starting at ZT0: a baseline day with spontaneous sleep permitted and a sleep deprivation day. On the sleep deprivation day, animals were prevented from sleeping from ZT0-ZT6 through gentle handling and the presentation of novel objects to encourage naturalistic exploration behaviour [34]. Each animal served as its own control for the effect of sleep deprivation and therefore the experimental unit in this study is an animal per recording day (n = 7).

### Vigilance state scoring and channel selection

EEG, LFP and EMG signals were used to score vigilance states in the Sleep Sign for Animals scoring environment (version 3.3.6.1602, SleepSign Kissei Comtec Co., Ltd., Nagano, Japan). Four second epochs were scored as WAKE, NREM or REM. Epochs with high frequency EEG and high amplitude EMG activity were scored as WAKE, epochs with a low frequency EEG characterised by delta band (0.5–4 Hz) slow waves and sigma band (11–15 Hz) spindles and a quiet EMG were scored as NREM sleep. Epochs with a wake-like EEG dominated by theta band activity and a quiet EMG were scored as REM sleep. Epochs with recording artefacts related to movement or electrostatic noise were rejected from further analyses in all channels (5.06 ± 0.19% of total recording time). Only vigilance states lasting ≥ 3 epochs were retained for further analysis to ensure clear differentiation of states. Channels with low MUA amplitude variation (i.e. without spiking activity) were rejected by visual inspection as were channels located in the corpus callosum (33/112 channels). For the purpose of inter-animal comparisons, each animal was represented by a single layer 5 channel.

### Low amplitude segment extraction

**Fig. 6:**
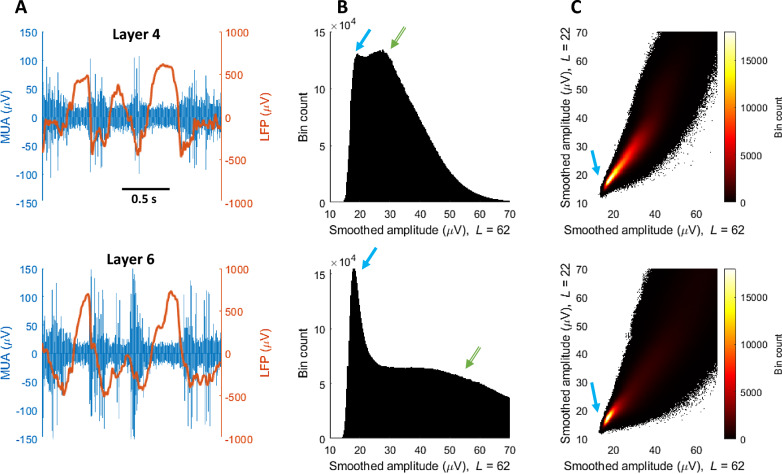
OFF period detection rationale. Top row depicts data from cortical layer 4, bottom row from layer 6. **A** MUA and LFP signal from different cortical layers from the same NREM sleep interval. OFF periods can be distinguished by a reduced MUA amplitude and often by the presence of LFP slow waves. The appearance of OFF periods, in terms of amplitude and duration, differs between and within layers. **B** 1D Histogram of NREM MUA amplitudes after Gaussian smoothing. L = length of smoothing window in ms, width factor = 2.5. The histogram of layer 6 has a bimodal distribution with a narrow low amplitude peak (blue arrow), which we call low amplitude (LA) data points, and a broad high amplitude peak (green arrow), which we interpret as non-LA period data points. The histogram of layer 4 is also bimodal but the peaks are closer together and have similar heights. In both cases, no obvious threshold exists at which to separate the peaks. **C** 2D histogram of MUA amplitude after Gaussian smoothing with two different window lengths. The histogram is unimodal with only the low amplitude peak retained (blue arrow). Rather than setting an amplitude threshold, LA data points can now be detected by finding points belonging to this high density region

The concept of distinct ON/OFF states necessitates that MUA recorded during NREM should be bimodally distributed. Assuming that OFF periods represent protracted periods of synchronised low amplitude activity (Fig. 6A), we smoothed the absolute values of MUA extracted from NREM sleep by convolution with a 62 ms Gaussian window (width factor = 2.5, sum of weights = 1) and plotted a 1D histogram of amplitudes. This generated the bimodal distribution upon which previous ‘threshold-crossing’ algorithms have been based [13, 22, 23] (Fig. 6B, top row). However, upon comparing different channels and recording periods we found it was not always clear where the distributions diverged (Fig. 6B, bottom row). As MUA amplitude during ON periods is more varied as a result of spiking events, we theorised that ON periods should be more sensitive to smoothing window length and that this property could be leveraged to facilitate differentiation of the distributions. We compared MUA amplitude smoothed with a 62 ms Gaussian window (width factor = 2.5, sum of weights = 1) and a shorter 22 ms Gaussian window (width factor = 2.5, sum of weights = 1) using a 2D histogram. Indeed, we consistently observed a dense region of low amplitude MUA points which we theorised may be reflecting OFF periods (Fig. 6C).

To explore this further, we sought to find the dimensions of this low amplitude region using Gaussian mixture modelling (GMM). A Gaussian mixture model is a probabilistic model that assumes all the data points are generated from a mixture of a finite number of multivariate Gaussian distributions or components. Unlike k-means clustering, these components and therefore the resulting clusters do not need to be spherical in shape. To find the parameters of the Gaussian components which maximize the likelihood of the model given the data, the two-step iterative Expectation–Maximization (EM) algorithm is employed. In the expectation step, the algorithm computes posterior probabilities of component memberships for each observation given the current parameter estimates. In the maximization step, the posterior probabilities from the previous step are used to re-estimate the model parameters by applying maximum likelihood. These steps are repeated until the change in loglikelihood function is less than the tolerance (${10}^{-5}$). Once the fitted GMM has been obtained, new points can be assigned to the component yielding the highest posterior probability (hard clustering).

The primary variable that must be input for GMM is the number of components (k) to fit from the data. When using k = 2 components, we unexpectedly found that the solution consistently overestimated the size of the low amplitude component. If this is indeed the data from OFF periods, this could suggest that the variation between types of ON period is greater than variation between OFF and ON periods. To resolve this, we decided to allow k to vary then select the resulting configuration that provides the optimal clustering solution. First, the smoothed MUA signals (L = 62 ms and 22 ms) from a subset of NREM episodes is clustered using GMMs with k = 1:8 components. Then, the optimal model is selected using a clustering evaluation index. Clustering evaluation indices are used to assess clustering performance when there is no ground truth, as is the case for binary OFF/ON period alternation. We investigated two such indices: the Calinsky-Harabasz index and the Davies-Bouldin index. The Calinsky-Harabasz index compares the dispersion within clusters with the dispersion between clusters whereas the Davies-Bouldin index compares the distance between clusters with the size of the clusters themselves. As expected, we found that the optimal clustering solution suggested by both methods produced more than 2 clusters. The lowest amplitude cluster tended to be much smaller than the equivalent with a 2-component model and more closely resembled the dense concentration of points in the MUA amplitude heatmap previously identified as the likely OFF period region (Fig. 6C). In the absence of clear differences in performance, we randomly selected the Calinsky-Harabasz index as our default.

**Fig. 7:**
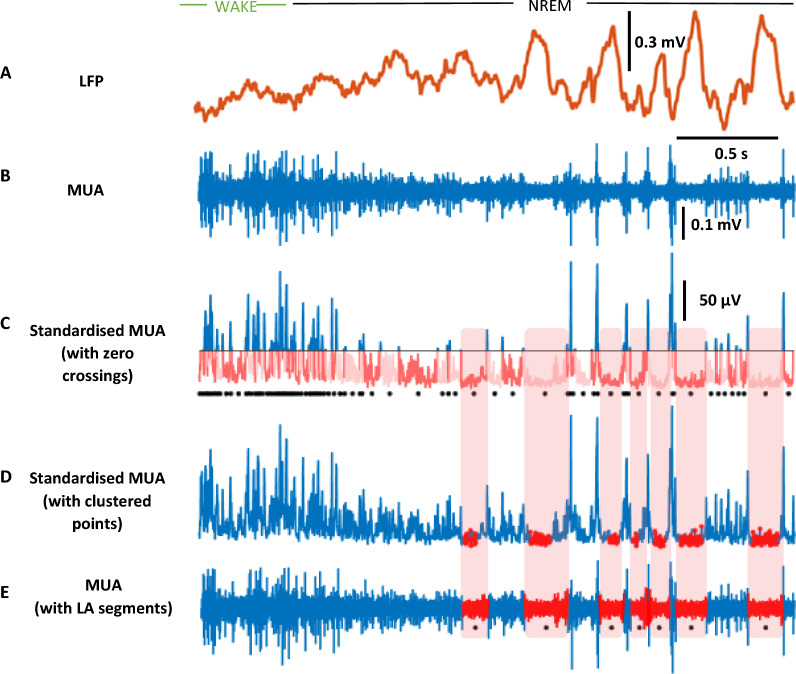
LA segment detection pipeline. Example of LA segment detection stages from a 3 s segment of layer 6 baseline day recording. **A** Local field potential (LFP) trace (0.5–100 Hz filtered). Note the appearance of slow waves following the transition from wake to non-REM sleep. **B** Raw decimated multi unit activity (MUA). **C** Standardised MUA whereby the raw signal is converted to absolute amplitude and then the mean of the absolute MUA during clean wake epochs from this 24 h recording is subtracted. The horizontal black line marks the new mean amplitude (0 μV). Each contiguous sequence of points below this line has been recoloured alternate shades of pink. These zero crossings represent the population of possible LA segments to be investigated. A black dot demarcates the centre of each zero crossing. **D** Standardised MUA with low amplitude cluster points recoloured red. **E** Raw MUA with final LA segments recoloured red. Final LA segments represent zero crossings which intersect with low amplitude cluster points. Black dots represent the centre of each LA segment

Although smoothing means that the data used for clustering is dependent on surrounding timepoints, the clustering step itself is independent of time. This contrasts with all existing descriptions of OFF periods which are understood as a feature observed in a linear time course of neuronal activity. To recapitulate the time domain, we decided to group low amplitude points into consolidated segments (Fig. 7A–E). First, we defined a population of time segments with below average MUA amplitude. This population was identified by taking all time segments in which the standardised MUA was below zero (Fig. 7C), where the standardised MUA is calculated by taking the absolute values of the MUA then subtracting the mean of these absolute values during WAKE epochs. The waking average was chosen to represent baseline MUA so that it would not be dependent on the number and duration of OFF periods in the signal. We then isolated those which coincided with at least one time point belonging to the low amplitude cluster (Fig. 7D). This final population of segments represents the low amplitude (LA) segments used during this analysis (Fig. 7E).

LA segment duration will inevitably depend to some degree on where the MUA amplitude threshold is set, increasing as the threshold is raised. There is a trade-off between setting the threshold low enough to register spiking activity but not so low as to register noise fluctuations in the MUA signal during neuronal silence. We looked at the sensitivity of LA segment duration to changes in this threshold and found that it is most stable when set at the mean amplitude of the MUA during wakefulness (Additional file 1: Figure S5), validating our threshold selection.

Due to their independent nature, a unique clustering solution and mean MUA amplitude was generated for each channel in each animal to detect LA segments. Where the same channel was measured over multiple days, we reapplied the configuration generated from the initial day under the assumption that these signals would be dependent.

Low amplitude segment extraction pipeline:

1. Cluster MUA signal smoothed at two window lengths (62 ms and 22 ms, NREM sleep only)
2. Detect lowest amplitude component
3. Assign smoothed MUA data to low amplitude cluster (all states)
4. Find negative zero-crossing half waves across all states (all states)
5. Re-assign clustered points to negative zero-crossings to find low amplitude segments

### Temporal, LFP phase and channel coherence analysis

The following temporal parameters of LA segments were assessed: incidence, average duration and total occupancy time per minute of each vigilance state (NREM/REM/WAKE) calculated from whole recordings or 30 min time bins on each recordings day.

For phase analyses, LFP signal was zero-phase distortion filtered in the delta band (0.5–4 Hz) to extract slow wave activity. The complex-valued analytic signal was then calculated using the Hilbert transform. The instantaneous phase angle in the interval [− *π*,*π*] for each element of the complex array was calculated by finding the inverse tangent and converted from radians to degrees. A phase of 0° corresponds to the peak of the oscillation and a phase of 180° to the trough of the oscillation. To measure the temporal coherence of LA segments between pairs of channels we generated the following statistic:

Pairwise channel coherence = (intercept(A|B)/sum(B) + intercept (B|A)/sum(A)) /2.

Where A and B are the time points of MUA signal within LA segments for two unique channels. A value of 0 denotes a situation in which all LA segments in both channels occur independently and a value of 1 denotes a situation in which all LA segments in both channels occur simultaneously. To estimate the random chance coherence between two channels we generated a randomly shuffled surrogate signal for each channel with an identical distribution of LA and non-LA segments. To achieve this, we fit a normally smoothed Kernel distribution to the distribution of LA and non-LA segment durations for each channel during the first hour of NREM sleep. We then randomly sampled these distributions in an alternating fashion to generate a shuffled sequence of LA and non-LA segments one hour in length. Only channels 1 to 12 were used in this analysis as in multiple animals the deepest 4 channels extended into the corpus callosum. In addition, due to channel rejection the maximum interchannel distance (11) was not available for all animals and was therefore omitted.

### Statistics

Statistical analyses were performed in MATLAB and R. Values are reported as mean ± standard error (SEM). The normality assumption of underlying distributions was assessed for each factor level by computing a Shapiro-Wilks test. Unless stated otherwise, significance of effects was tested using one- or two-way repeated measure ANOVAs (within-subject factors “Vigilance state [NREM, REM, WAKE]”, “Day [baseline, SD]” and/or “Time”) with animal ID as a factor followed by post-hoc pairwise t-tests with Bonferroni correction. Circular statistics for phase analysis were performed using the CircStat toolbox [35]. Non-uniformity of each distribution against the von Mises distribution was confirmed using a Rayleigh test for circular data. Differences in mean direction were tested using a parametric Watson-Williams multi-sample test for equal means with Bonferroni correction. Statistical significance in all tests was considered as p < 0.05. For box plots, the middle, bottom, and top lines correspond to the median, bottom, and top quartile, and whiskers to lower and upper extremes minus bottom quartile and top quartile, respectively.

### GUI design

The LA segment detection algorithm was incorporated into a MATLAB program with a user-friendly GUI that allows the detection of LA segments in new data sets, provides a visual representation of the results and generates a range of useful summary statistics (https://github.com/sjoh4302/OFFAD). Furthermore, this GUI allows for post-processing of LA segments, such as removal of brief interruptions, to fit individual user expectations of OFF periods.

## Supplementary Information

**Additional file 1: Figure S1.** LA segment occupancy (A) and duration (B) on baseline day across light period (ZT0-ZT12). N=7. Mean ± SEM. **Figure S2.** Summary statistics for linear regression of LA incidence (relative to ZT 6) against zeitgeber time for each LA segment duration category. Df = degrees of freedom. **Figure S3.** All original channel coherence matrices (N=7). **Figure S4.** All surrogate channel coherence matrices (N=7). **Figure S5.** Sensitivity of LA segment duration to MUA amplitude threshold. Average LA segment duration detected in a layer 5 channel from a baseline recording day in a single mouse as a function of MUA amplitude threshold at step 4 in the LA segment detection pipeline where 100% (denote with a vertical dashed line) corresponds to the average MUA amplitude measured during wakefulness (blue). As the threshold is raised, LA segment duration increases. The first derivative of this curve is plotted on a separate axis (orange). Note that other than when the threshold is set at such a low point as to miss most LA segments, the first derivative is lowest at 100%, suggesting that LA segment duration is least sensitive when the threshold is set to the average MUA amplitude measured during wakefulness.

## Data Availability

Code for OFF period detection (OFFAD) is available on GitHub (https://github.com/sjoh4302/OFFAD). The datasets during and/or analysed during the current study available from the corresponding author on reasonable request.
