# Supplementary material for: Detection of neuronal OFF periods as low amplitude neural activity segments
Source: BMC Neurosci. 2023 Feb 21;24:13. doi: 10.1186/s12868-023-00780-w (PMC9942432; doi:10.1186/s12868-023-00780-w)

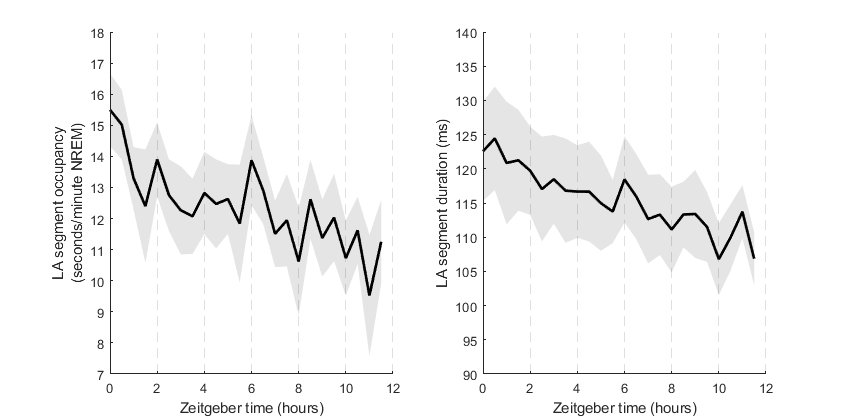


Figure S1: LA segment occupancy (A) and duration (B) on baseline day across light period (ZT0-ZT12). N=7. Mean ± SEM.

| Category | Df (numerator) | Df (denominator) | F statistic | P value |
| --- | --- | --- | --- | --- |
| <50ms | 1 | 70 | 0.13293 | 0.71651 |
| 50-100ms | 1 | 82 | 14.862 | 0.00022902 |
| 100-150ms | 1 | 82 | 24.497 | 3.9123e-06 |
| 150-200ms | 1 | 82 | 84.347 | 3.0974e-14 |
| 200-250ms | 1 | 82 | 167.58 | 1.6168e-21 |
| 250+ms | 1 | 82 | 104.5 | 2.7193e-16 |

Figure S2: Summary statistics for linear regression of LA incidence (relative to ZT 6) against zeitgeber time for each LA segment duration category. Df = degrees of freedom.


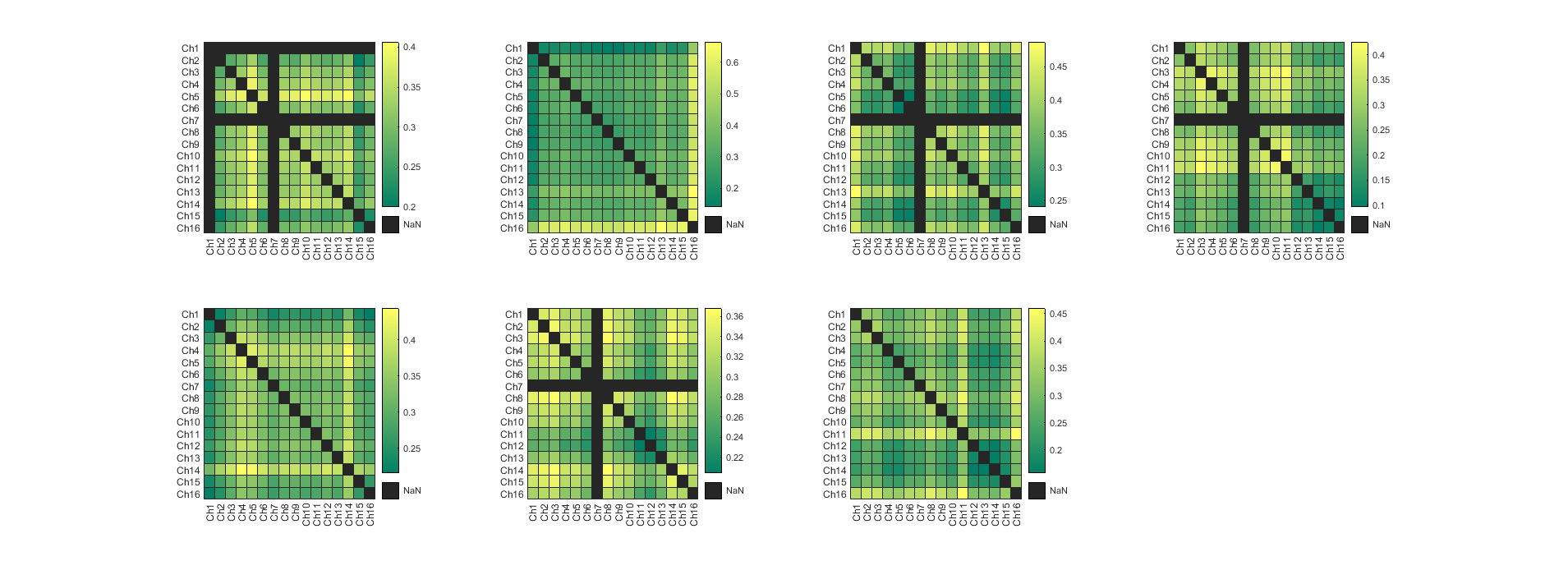


Figure S4: All surrogate channel coherence matrices (N=7).


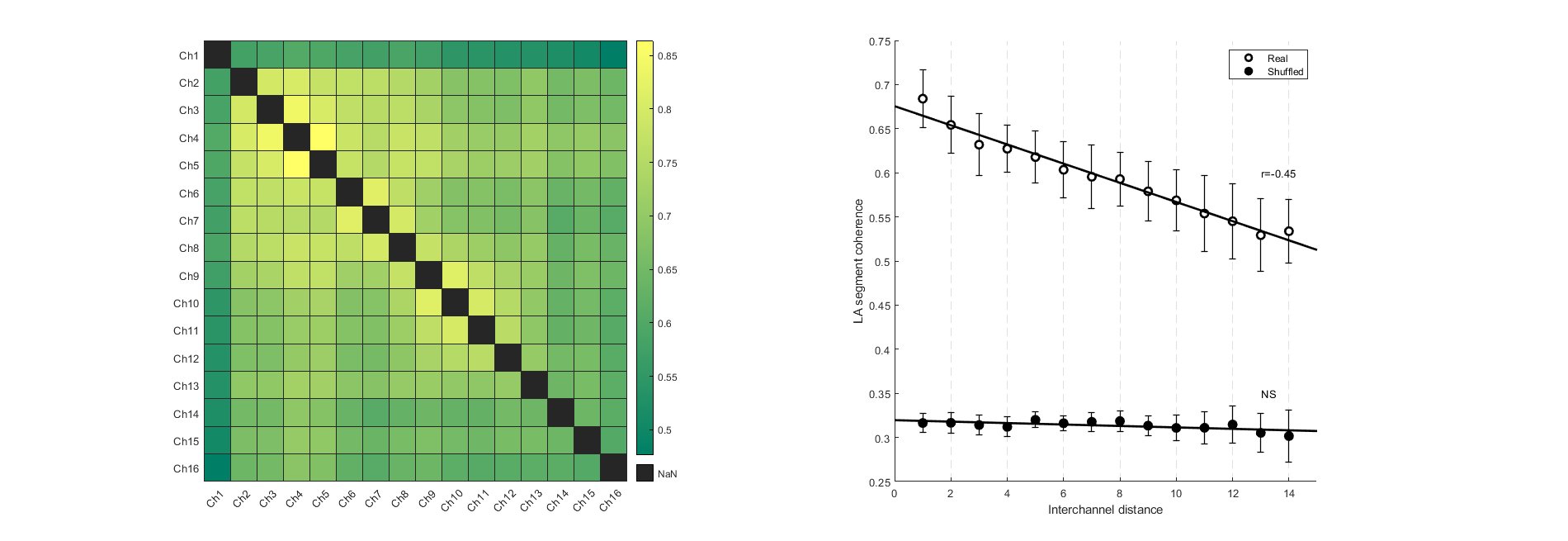

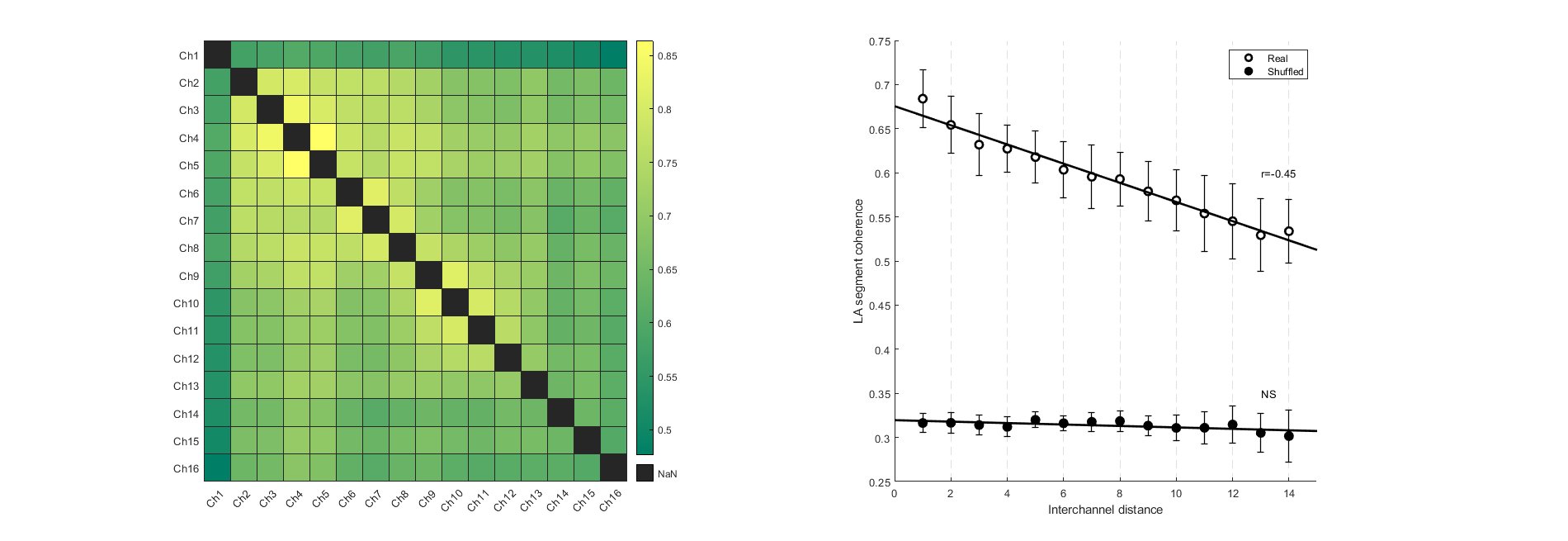

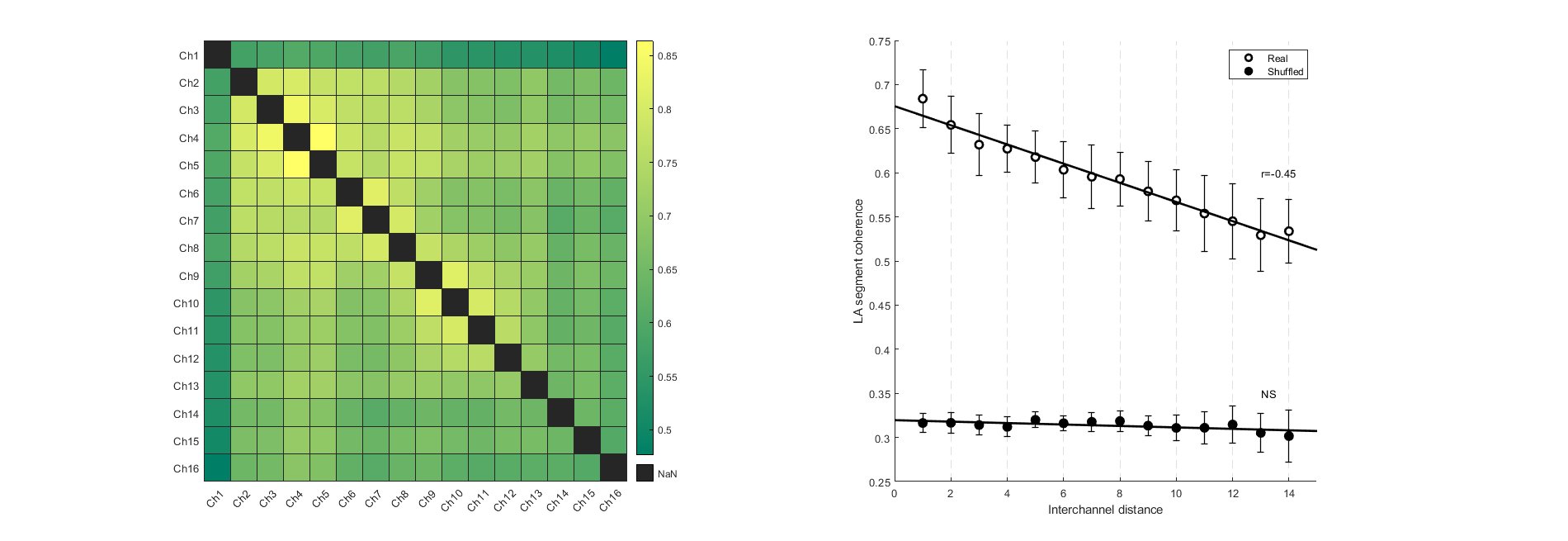

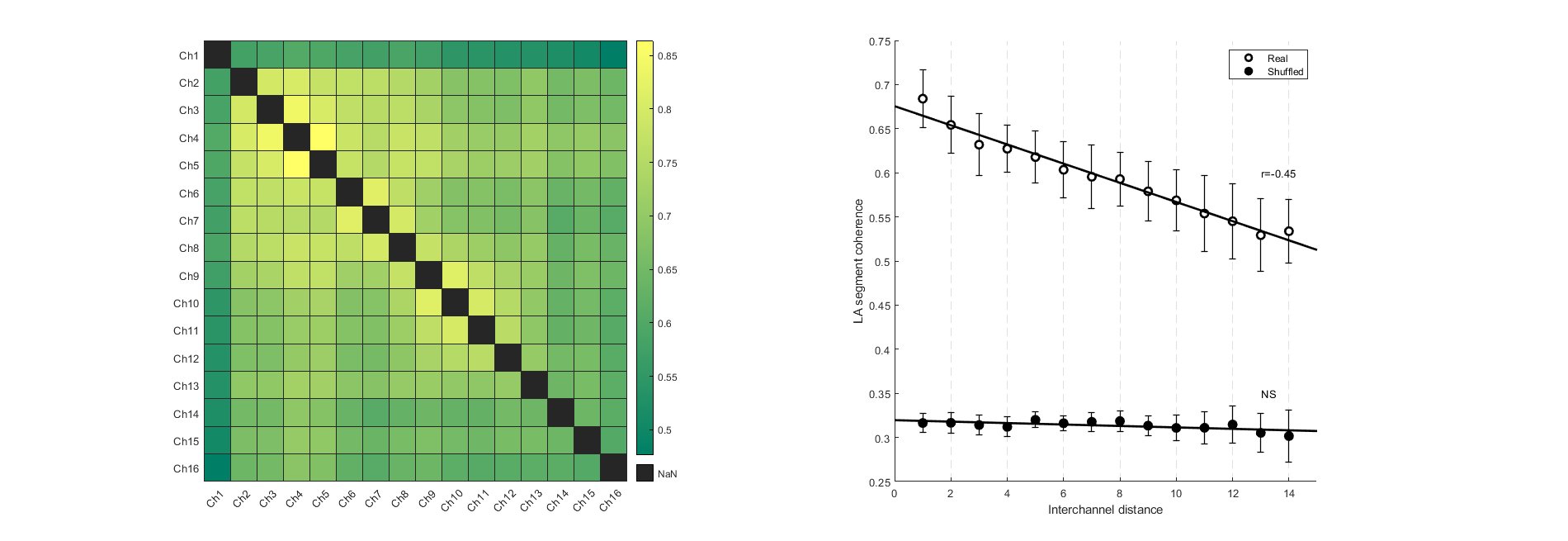

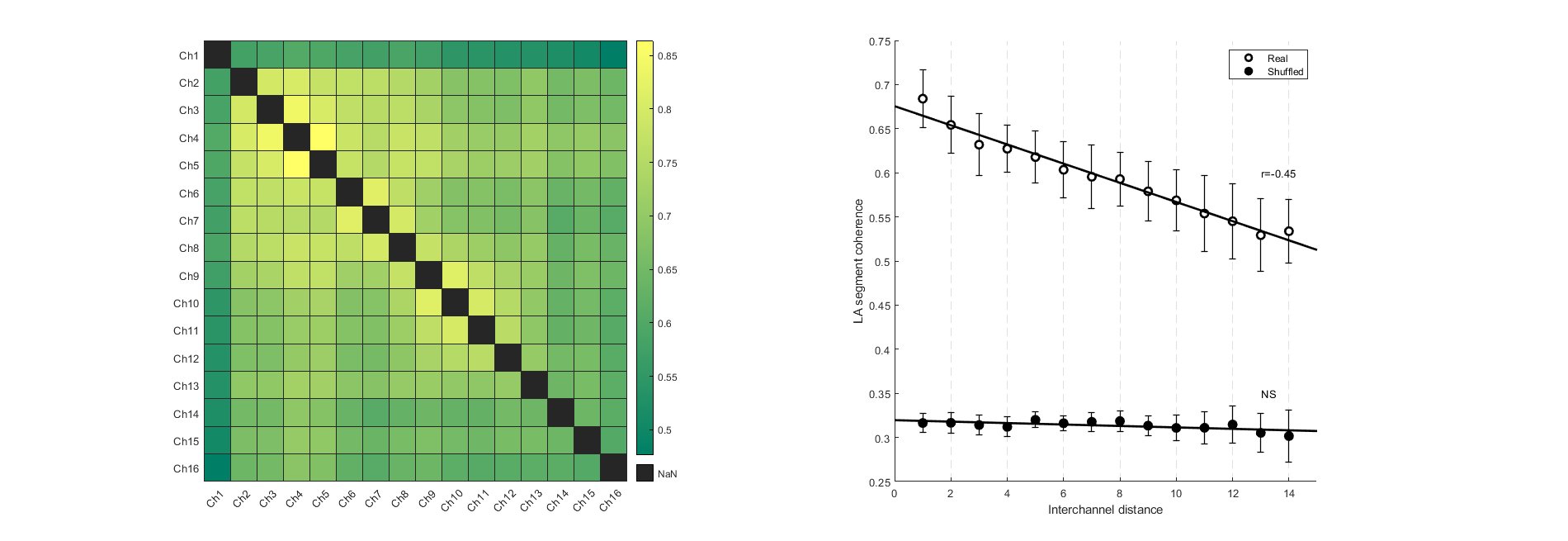

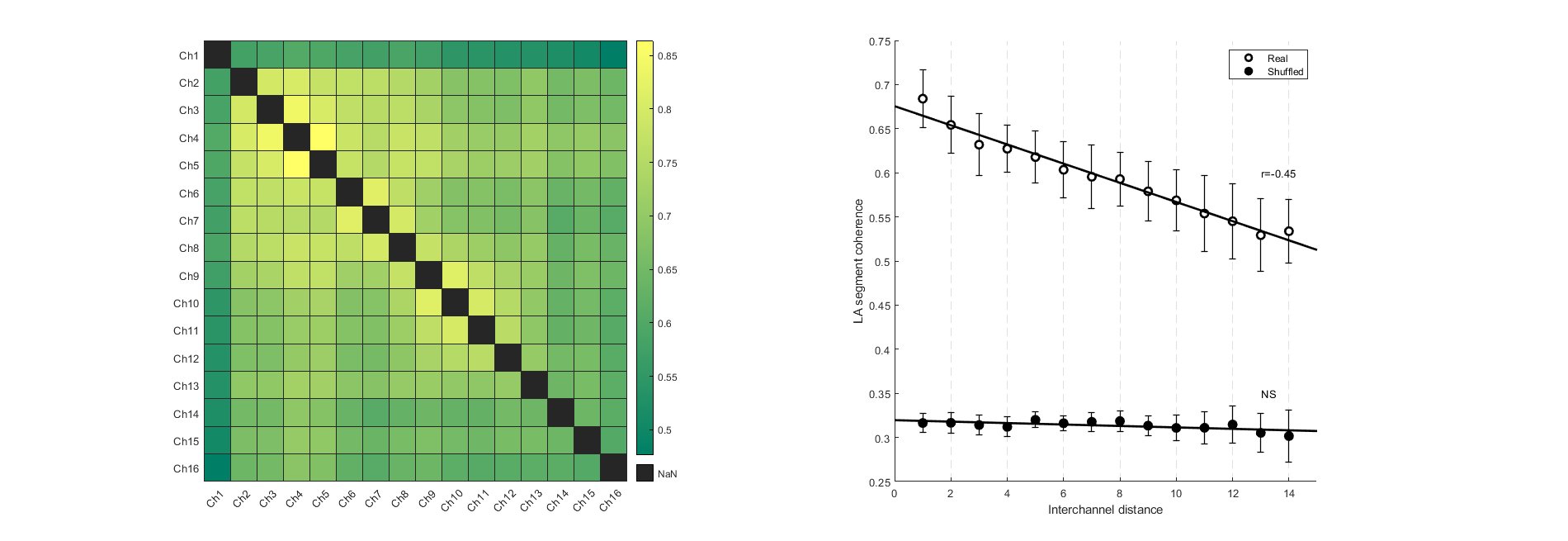

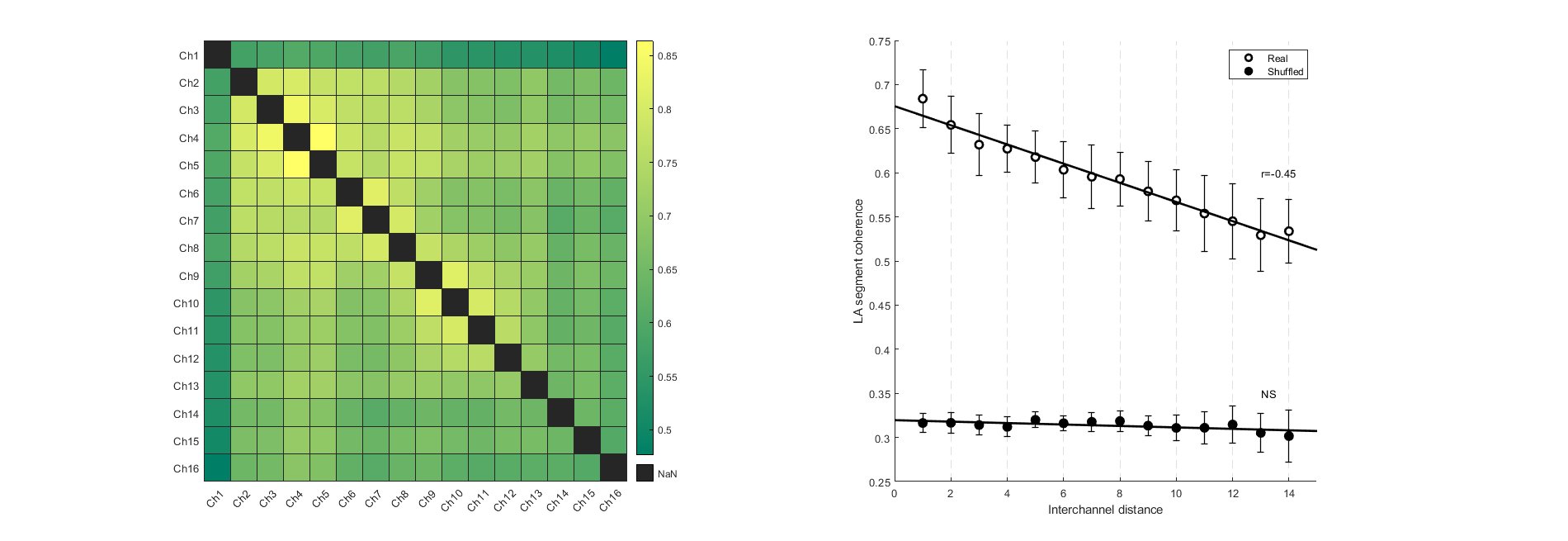

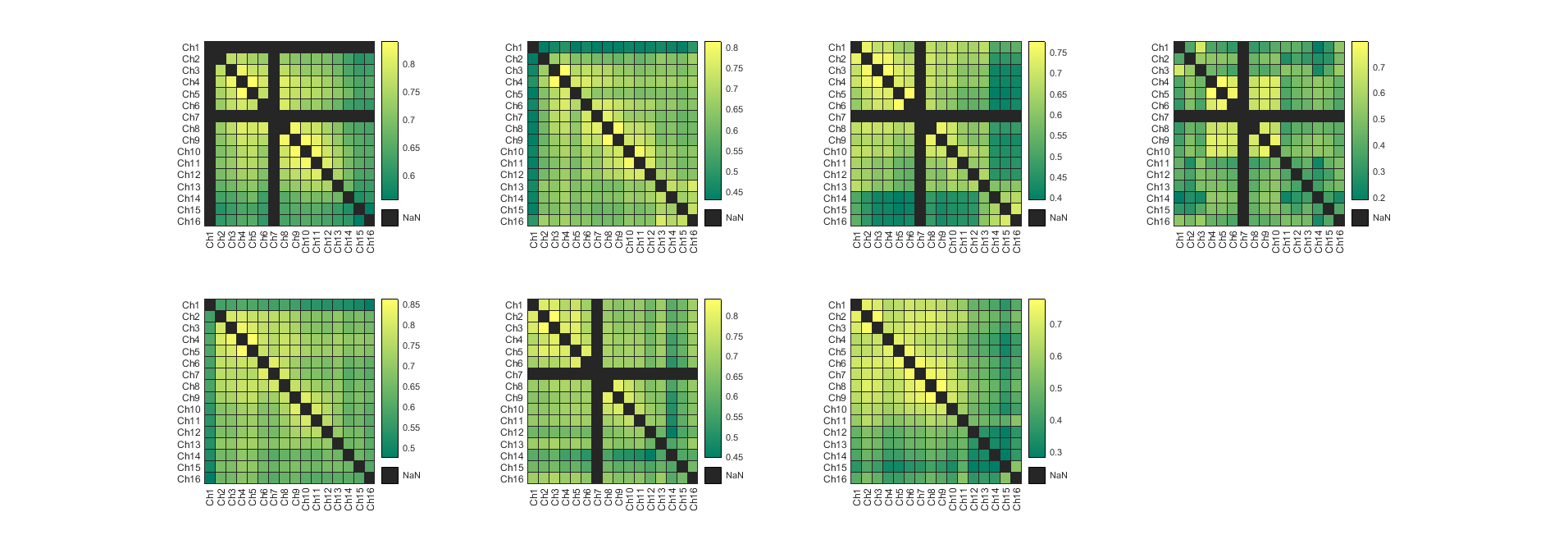


Figure S3: All original channel coherence matrices (N=7).


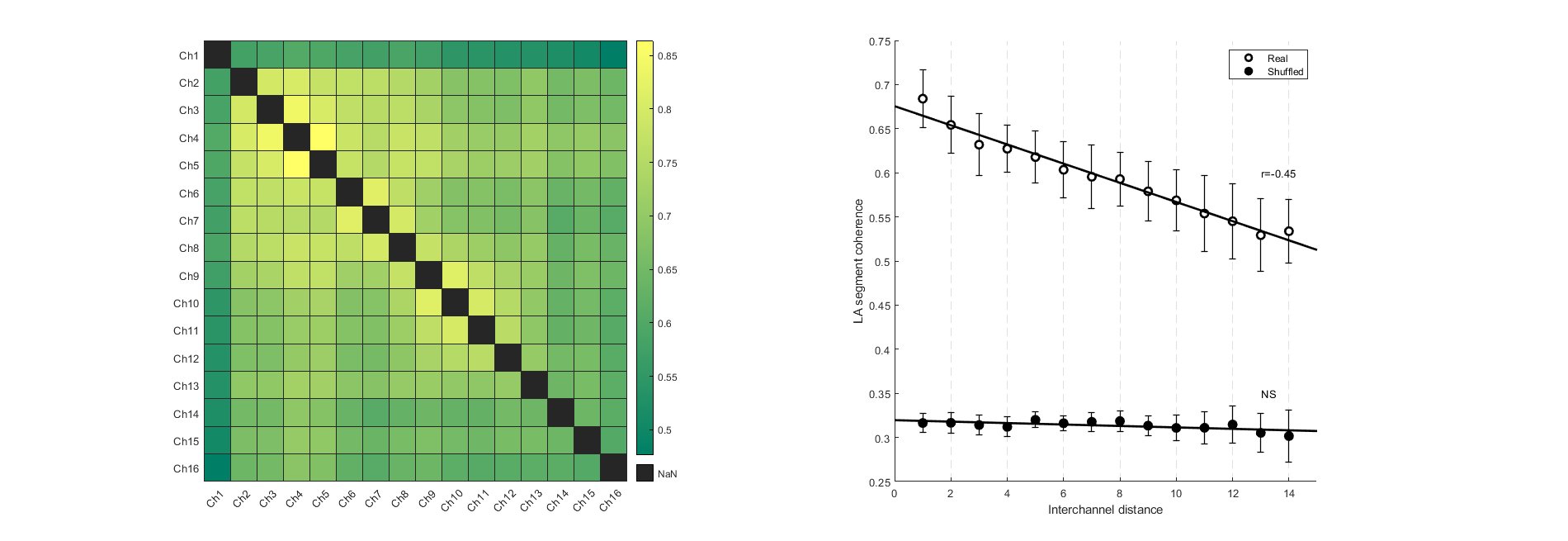

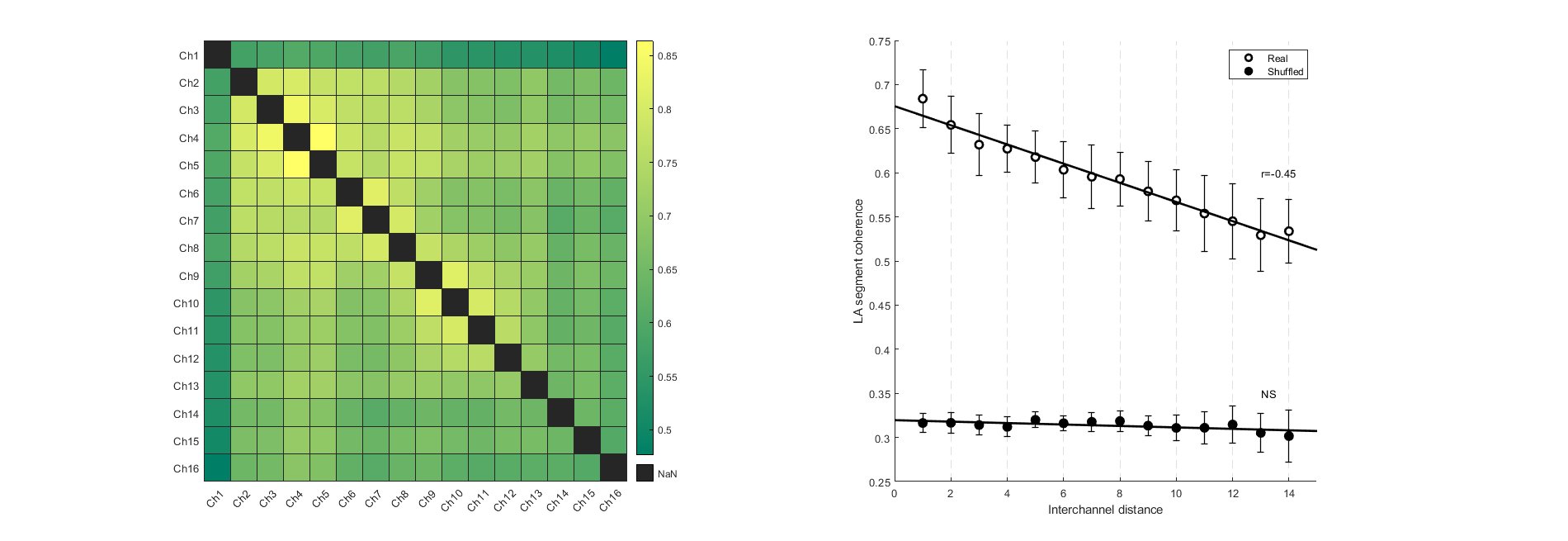

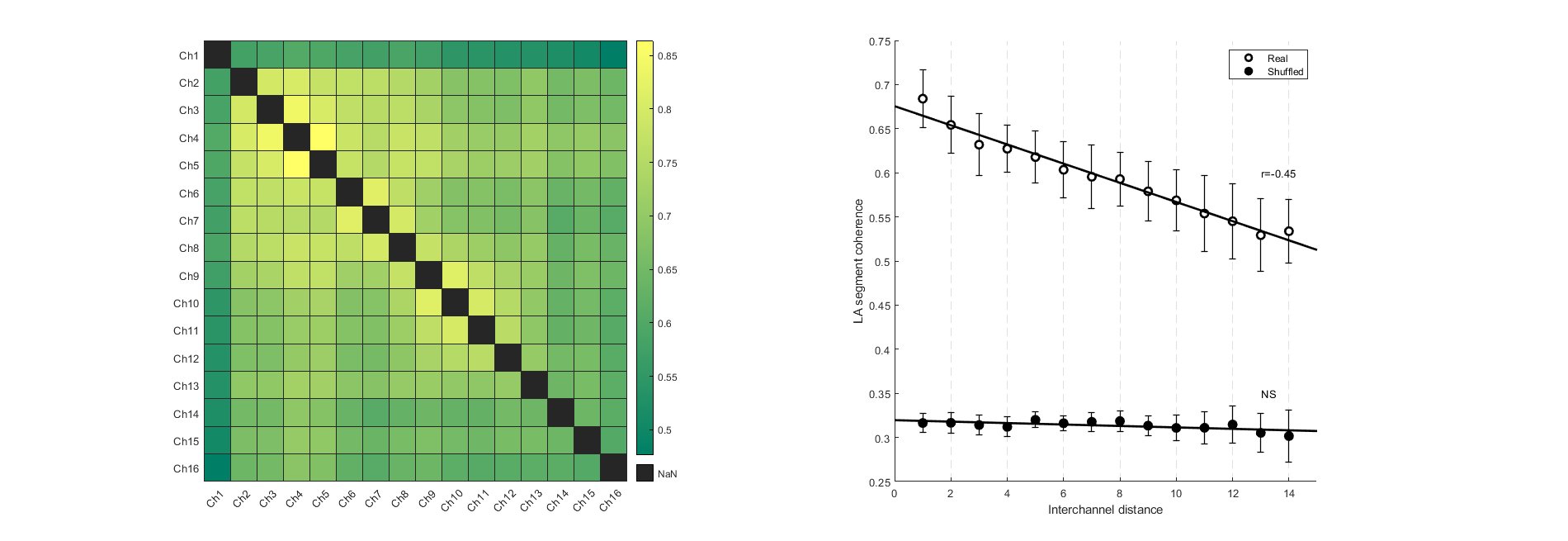

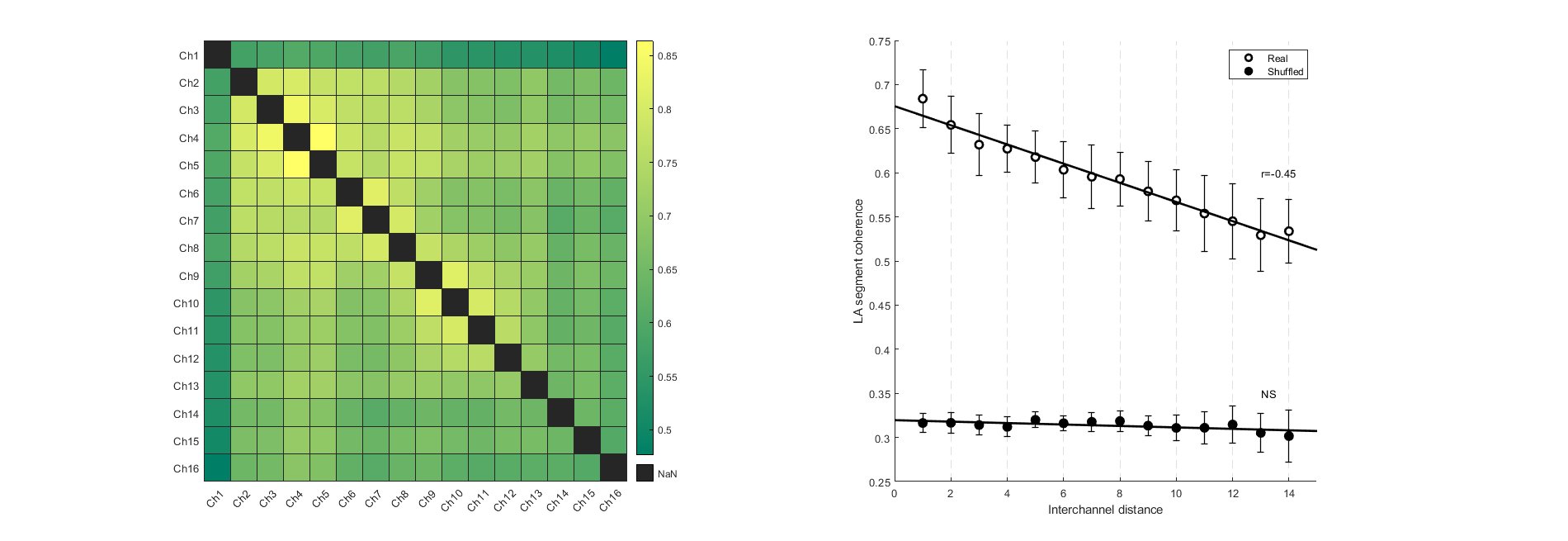

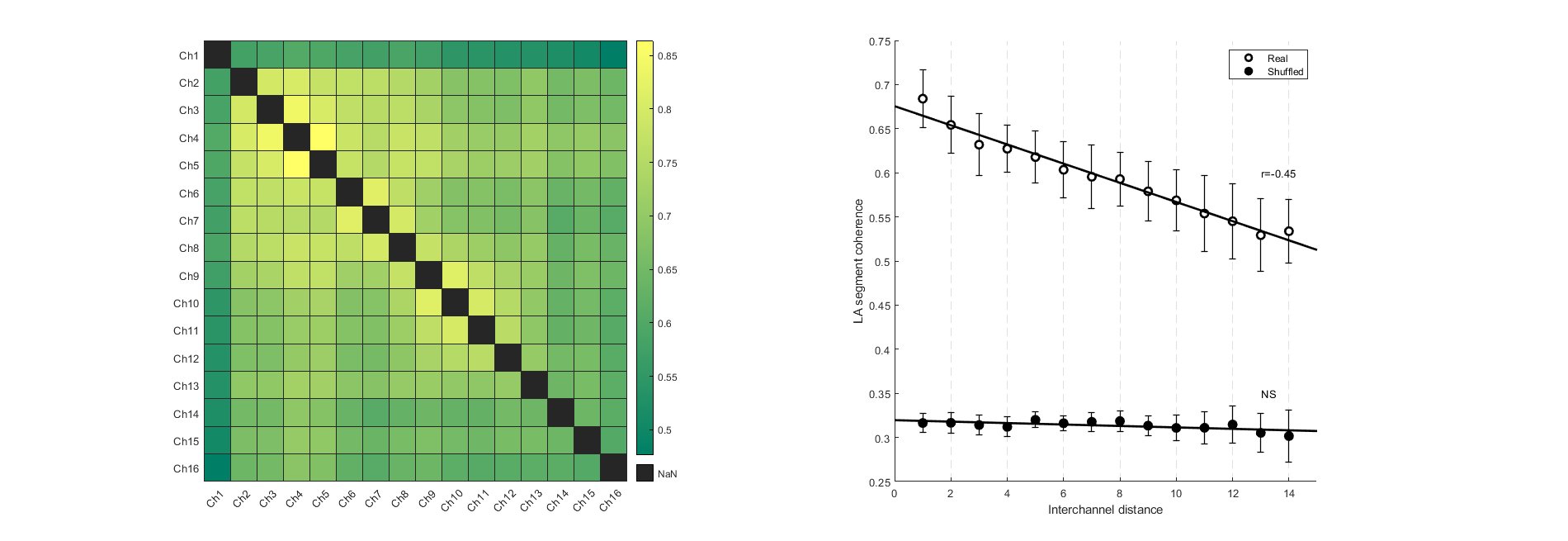

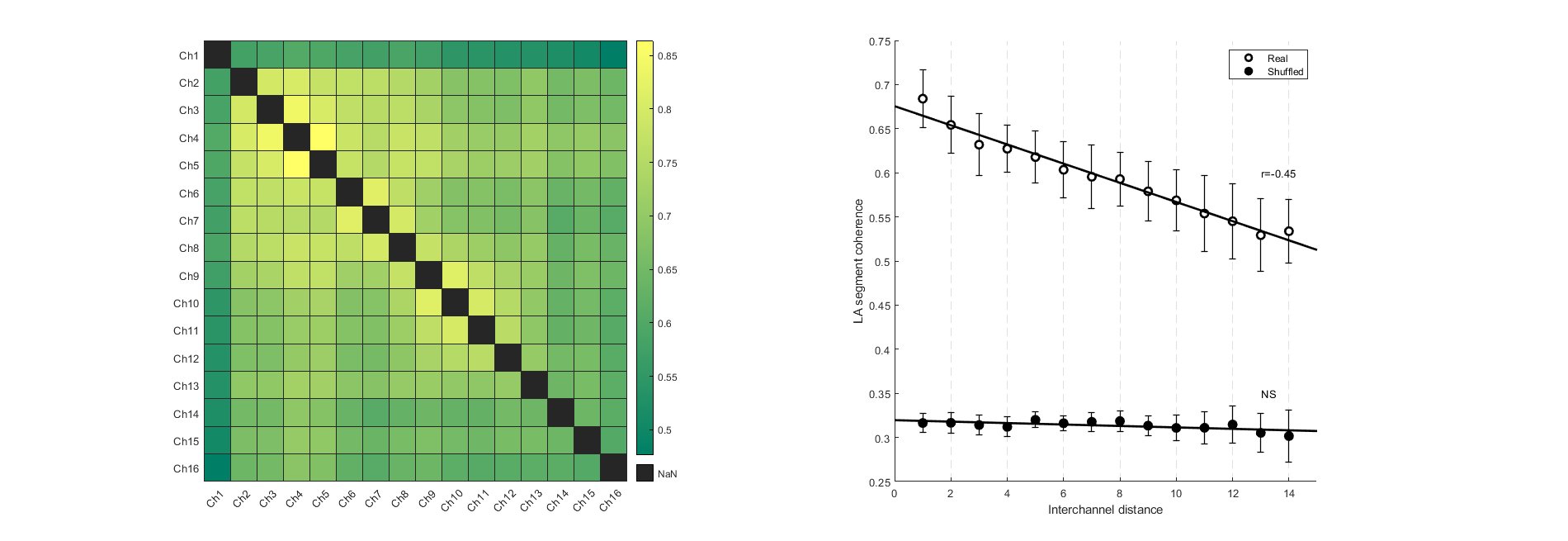

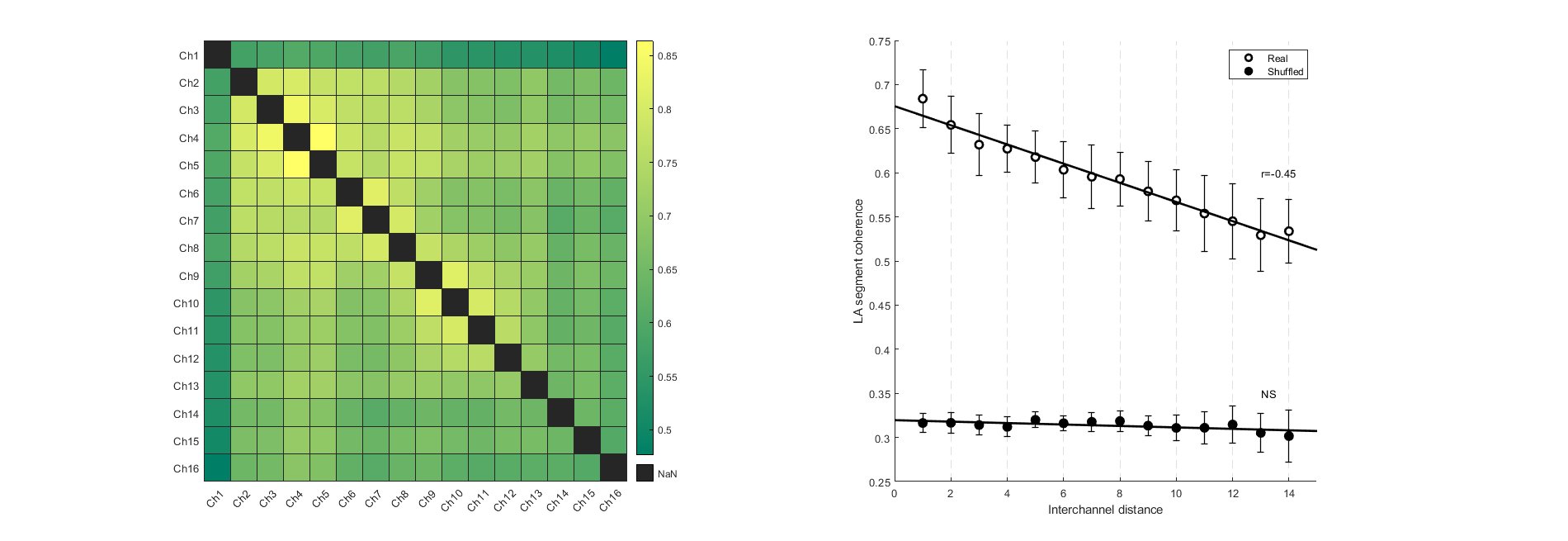


figure S5: Sensitivity of LA segment duration to MUA amplitude threshold. Average LA segment duration detected in a layer 5 channel from a baseline recording day in a single mouse as a function of MUA amplitude threshold at step 4 in the LA segment detection pipeline where 100% (denote with a vertical dashed line) corresponds to the average MUA amplitude measured during wakefulness (blue). As the threshold is raised, LA segment duration increases. The first derivative of this curve is plotted on a separate axis (orange). Note that other than when the threshold is set at such a low point as to miss most LA segments, the first derivative is lowest at 100%, suggesting that LA segment duration is least sensitive when the threshold is set to the average MUA amplitude measured during wakefulness.


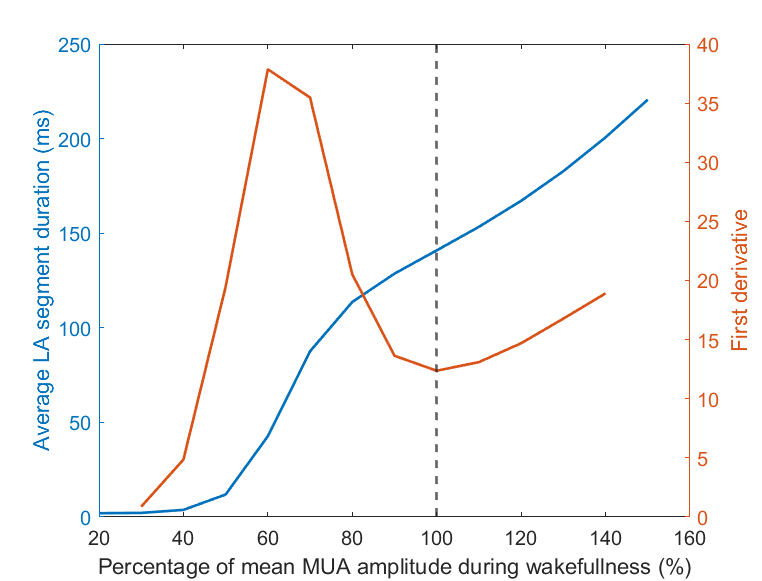

Supplement: Supplementary file 1 — Additional file 1: Figure S1. LA segment occupancy (A) and duration (B) on baseline day across light period (ZT0-ZT12). N=7. Mean ± SEM. Figure S2. Summary statistics for linear regression of LA incidence (relative to ZT 6) against zeitgeber time for each LA segment duration category. Df = degrees of freedom. Figure S3. All original channel coherence matrices (N=7). Figure S4. All surrogate channel coherence matrices (N=7). Figure S5. Sensitivity of LA segment duration to MUA amplitude threshold. Average LA segment duration detected in a layer 5 channel from a baseline recording day in a single mouse as a function of MUA amplitude threshold at step 4 in the LA segment detection pipeline where 100% (denote with a vertical dashed line) corresponds to the average MUA amplitude measured during wakefulness (blue). As the threshold is raised, LA segment duration increases. The first derivative of this curve is plotted on a separate axis (orange). Note that other than when the threshold is set at such a low point as to miss most LA segments, the first derivative is lowest at 100%, suggesting that LA segment duration is least sensitive when the threshold is set to the average MUA amplitude measured during wakefulness. [file 12868_2023_780_MOESM1_ESM.docx]
